# Protozoan Neglected Tropical Diseases (NTDs) Target Inhibition of Alkaloids from *Croton linearis* Jacq Leaves: A Molecular Docking and ADMET Approach

**DOI:** 10.3390/ph18111715

**Published:** 2025-11-12

**Authors:** Julio A. Rojas-Vargas, Jesús García-Díaz, Julio César Escalona-Arranz, Jakub Chlebek, Lianet Monzote, William N. Setzer, Juan A. Castillo-Garit

**Affiliations:** 1 Doctorado en Informática Aplicada a Salud y Medio Ambiente, Universidad Tecnológica Metropolitana, Ignacio Valdivieso 2409, San Joaquín, Santiago 8940577, Chile; 2 Departament of Chemistry, Exact and Natural Science Faculty, Universidad de Oriente, Santiago de Cuba 90500, Cuba; 3 Department of Pharmacognosy and Pharmaceutical Botany, Faculty of Pharmacy in Hradec Kralove, Charles University, Akademika Heyrovského 1203, 500 05 Hradec Kralove, Czech Republic or garciadje@faf.cuni.cz (J.G.-D.); chlej2aa@faf.cuni.cz (J.C.); 4 Department of Pharmaceutical Sciences, Faculty of Sciences, Universidad Católica del Norte, Angamos 0610, Antofagasta 124 0000, Chile; julio.escalona@ucn.cl; 5 Department of Parasitology, Institute of Tropical Medicine “Pedro Kourí”, Marianao 13, La Habana 10400, Cuba; monzote@ipk.sld.cu; 6 Research Network Natural Products Against Neglected Diseases (ResNetNPND), 48149 Munster, Germany; wsetzer@chemistry.uah.edu; 7 Aromatic Plant Research Center, 230 N 1200 E, Suite 100, Lehi, UT 84043, USA; 8 Department of Chemistry, University of Alabama in Huntsville, Huntsville, AL 35899, USA; 9 Instituto Universitario de Investigación y Desarrollo Tecnológico (IDT), Universidad Tecnológica Metropolitana, Ignacio Valdivieso 2409, San Joaquín, Santiago 8940577, Chile

**Keywords:** molecular docking, neglected tropical diseases, *Croton linearis* Jacq, alkaloids, multitarget inhibitors, protozoan enzymes

## Abstract

**Background/Objectives:** Neglected tropical diseases (NTDs) caused by protozoan parasites such as *Trypanosoma cruzi*, *Trypanosoma brucei*, *Leishmania* spp., and *Plasmodium falciparum* remain a global health challenge due to limited therapies and increasing drug resistance. Natural products provide diverse scaffolds for antiparasitic drug discovery. This study aimed to investigate the multitarget inhibitory potential of alkaloids isolated from *Croton linearis* Jacq. against validated protozoan enzymes. **Methods:** Eighteen alkaloids were virtually screened against 17 molecular targets relevant to protozoan parasites. Protein–ligand docking simulations were performed using crystallographic structures of enzymes, including Cyp51, DHFR-TS, PTR1, AD-kinase, and DHODH. Predicted interactions were analyzed to identify hydrogen bonds, hydrophobic contacts, and π–π stacking with key residues in the active sites. **Results:** Several alkaloids exhibited high binding affinities, in some cases surpassing co-crystallized ligands. Reticuline, norsalutaridine, laudanosine, and jacularine consistently showed the strongest activity, with docking scores ranging from −8.0 to −9.3 kcal/mol across multiple targets. Notably, norsalutaridine displayed the highest predicted affinity for *L. infantum* Cyp51, while reticuline showed strong binding to *T. cruzi* DHFR-TS and *L. major* PTR1. **Conclusions:** The study highlights the potential of *C. linearis* alkaloids as multitarget inhibitors against protozoan parasites. These compounds represent promising lead candidates for the development of antiparasitic agents, while emphasizing the value of natural product scaffolds for neglected disease drug discovery. The findings also support the future exploration of semisynthetic derivatives to optimize activity and selectivity.

## 1. Introduction

According to the World Health Organization (WHO), Neglected Tropical Diseases (NTDs) are a group of diseases that mainly affect the poorest regions of the world [1]. They are in the tropical zone of the planet and arouse limited commercial interest on the part of large pharmaceutical companies, since many of these diseases are practically nonexistent in the richer regions of the world. Currently, nearly one billion individuals, i.e., one sixth of the world’s population, are affected by some of these diseases, most of them concentrated in Africa and Latin America. Many of these diseases are also associated with a lack of hygiene in the population, which in turn is related to a lack of education, something very common in the poorest regions of the world. Currently, some 17 diseases are considered as NTDs, many of which are not well known. They are characterized by being endemic and cannot spread outside tropical regions; for example, dracunculiasis (Guinea worm), American trypanosomiasis (Chagas disease), African trypanosomiasis (sleeping sickness), dengue, malaria, leishmaniasis, leprosy, lymphatic filariasis, and schistosomiasis, among others.

NTDs represent a significant health burden in large parts of the world. Some *Croton* species are notable for their production of alkaloids, which are biogenetically related to benzylisoquinolines, such as morphinandienones, apomorphine, and tetrahydroprotoberberine alkaloids [2,3]. *Croton linearis* Jacq is an evergreen shrub that grows in Caribbean regions (Cuba, Jamaica), the Bahamas, and Florida [4]. In the Bahamas, the crushed leaves have a small effect on bronchial and head colds when inhaled, owing to the release of fragrant oils. Tea eases symptoms of colds, flu, and influenza. Also, the decoction of leaves is used by oral route or in baths to relieve pains related to the menstrual period, after birth, and rheumatism [4]. Asprey and Thornton reported that it is usually used by Jamaican peasants as a hair wash, as in Browne’s day, it is still used in baths for fever and colds, for the treatment of which a tea made with the plant may also be taken [5].

Alkaloids have been documented as major components for this plant. The studies conducted in Jamaica by Haynes, Farnsworth et al. in the 1960s report the isolation of benzylisoquinoline type alkaloids hernovine, nuciferine, wilsonirine, jacularine, crotonosine, and linearisine, as well as morphinandienone type alkaloids 8,14-dihydrosalutaridine and 8,14-dihydronorsalutaridine. Recently Garcia et al. [6], reported six alkaloids isolated from *C. linearis* leaves: laudanidine, laudanosine, reticuline, corydine, glaucine, and cularine. Recent studies on *C. linearis* have demonstrated its significant antiprotozoal potential, particularly against *Leishmania* species, while revealing negligible antibacterial or antifungal activity. For instance, the essential oil derived from *C. linearis* stems, featuring major components such as 1,8-cineole, α-pinene, and epi-γ-eudesmol, exhibited potent in vitro efficacy with IC_50_ values of 21.4 μg/mL (promastigotes) and 18.9 μg/mL (amastigotes) of *Leishmania amazonensis*, alongside acceptable selectivity indexes of 2–3. Moreover, molecular docking studies revealed that epi-γ-eudesmol interacts effectively with *Leishmania* target enzymes, supporting its mechanistic plausibility [7].

Additionally, isoquinoline alkaloids isolated from *C. linearis* leaves, namely reticuline, laudanidine, and 8,14-dihydrosalutaridine, displayed moderate antiplasmodial activity against chloroquine-resistant *Plasmodium falciparum* K1 strains, with IC_50_ values of 46.8 ± 0.6, 17.7 ± 0.6, and 16.0 ± 0.5 μM, respectively, and no cytotoxicity up to 64 μM [8]. This underscores the versatility of alkaloids as antiparasitic scaffolds, particularly for malaria. Further supporting evidence comes from bioassay-guided fractionation of *C. linearis* leaf extracts, which revealed IC_50_ values in the range of 1–26 μg/mL against various protozoa, including *Leishmania infantum*, *Trypanosoma cruzi*, and *Leishmania* species, thereby, substantiating the antiprotozoal efficacy of both crude extracts and isolated alkaloids [7]. Together, these findings suggest that *C. linearis* produces a diverse chemical arsenal of monoterpenoid-rich essential oils and isoquinoline alkaloids with selective antiparasitic activity. Such evidence supports the hypothesis that these compounds could serve as valuable lead structures for the design and synthesis of novel semisynthetic antiprotozoal agents.

Natural products have long been recognized as valuable sources of bioactive com-pounds with therapeutic potential, especially in the search for new treatments for NTDs. A variety of studies have consistently underscored the significance of their diverse chemical structures and biological activities, which position them as optimal candidates for the identification of new pharmaceutical agents. Although conventional in vitro methods re-main paramount for evaluating antiparasitic effects, the integration of computational modeling has become progressively advantageous for predicting molecular targets and elucidating potential mechanisms of action at the nascent stages of research. Natural products have long been recognized as valuable sources of bioactive compounds with therapeutic potential, especially in the search for new treatments for NTDs. Numerous studies continue to highlight their diverse chemical structures and biological activities, which make them ideal candidates for drug discovery. While traditional in vitro approaches remain important for assessing antiparasitic effects, the incorporation of computational modeling has become increasingly advantageous for predicting molecular targets and understanding potential mechanisms of action at earlier stages of research [9].

Comprehensive phytochemical reviews further emphasize the importance of integrating computational tools into natural product research. The wide range of pharmaco-logical activities observed in plant-derived compounds suggests that many of these metabolites may interact with multiple biological pathways relevant to NTDs. Molecular modeling approaches, including virtual screening and docking simulations, provide valuable insights into these complex interactions, supporting the selection of promising com-pounds for further pharmacological development [10].

The growing use of artificial intelligence and cheminformatics has significantly enhanced the efficiency of natural product-based drug discovery. These advanced technologies allow researchers to analyze large datasets, predict bioactivity and toxicity profiles, and accelerate the identification of drug-like candidates. As highlighted in recent work, such approaches help streamline the discovery process by complementing traditional methods and enabling a more targeted exploration of nature-derived chemical diversity [11]. Molecular docking is a modeling technique to study how a small molecule (ligand/drug) interacts with a macro-molecule (protein/enzyme, DNA, etc.). This molecular modeling technique basically addresses three key goals—virtual screening, pose prediction, and binding affinity estimation. Taking into account the in vitro antiprotozoal potentialities showed by extract of *C. linearis*, which different alkaloids were identified, in this work, we have studied the potential bio-chemical interaction of identified alkaloids from *C. linearis* leaves on different protozoan NTDs targets using a molecular docking approach to suggest possible mechanisms of these metabolites.

## 2. Results and Discussion

**Figure 1:**
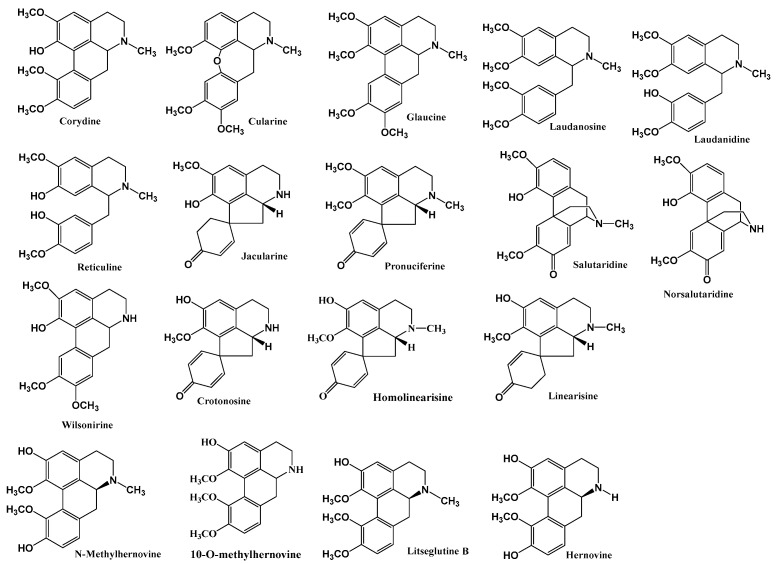
Chemical structure of alkaloids isolated from *Croton linearis* Jacq leaves.

To explore the therapeutic potential of natural alkaloids derived from *C. linearis*, a comprehensive molecular docking study was conducted against a panel of validated enzymatic targets implicated in various NTDs, including leishmaniasis, Chagas disease, sleeping sickness, and malaria [10]. The selection criteria for these alkaloids (Figure 1) are based on the previously reported in vitro antiprotozoal activities of *C. linearis* extracts [8,12]. The selected alkaloids were docked into the active sites of key parasite enzymes using a structure-based approach to predict their binding affinity and interaction profiles.

The results revealed notable differences in binding energies across targets, with several alkaloids demonstrating comparable or superior affinity to that of co-crystallized reference ligands. These findings provide valuable insights into the structure-activity relationships of these compounds and support their potential as lead molecules for the development of novel antiparasitic agents.

To assess the inhibitory potential of alkaloids isolated from *C. linearis* against protozoan targets implicated in NTDs, molecular docking was performed against a panel of enzymes from *Trypanosoma* (Table 1), *Leishmania*, and *Plasmodium* species (Table 2). The binding affinity (Δ*G*_dock_) values revealed that norsalutaridine, reticuline, and salutardine consistently displayed strong interactions across multiple targets, with particularly high affinity for *Leishmania infantum* Cyp51 (−9.3, −8.8, and −9.0 kcal/mol, respectively), outperforming the co-crystallized ligand TPF (−8.0 kcal/mol).

These alkaloids also exhibited favorable docking scores against *T. cruzi* DHFR-TS and *T. brucei* ADKinase. Reticuline, for instance, reached −8.5 kcal/mol in DHFR-TS and −8.1 kcal/mol in ADKinase, indicating potential multitarget activity. Such results support the idea that benzylisoquinoline alkaloids may interfere with multiple metabolic pathways, enhancing their pharmacological relevance.

Among the tested alkaloids from *C. linearis*, reticuline, salutaridine, and norsalutaridine emerged as top candidates across multiple enzyme targets from *L. infantum*, *T. cruzi*, *T. brucei*, and *P. falciparum*. Against *L. infantum* Cyp51, the most promising target for leishmaniasis, norsalutaridine achieved the best docking score (−9.3 kcal/mol), outperforming both reticuline (−8.8) and the co-crystallized ligand TPF (−8.0). This suggests a strong and specific interaction, potentially driven by aromatic stacking with the heme group and polar contacts within the enzyme’s hydrophobic pocket.

Similarly, reticuline demonstrated superior binding to *T. cruzi* DHFR-TS (−8.4) and *T. brucei* ADKinase (−8.1), indicating its broad inhibitory potential.

For PTR1 from *L. major*, wilsonirine demonstrated the highest docking score (−8.9), exceeding the reference ligand (−7.9). This compound’s extended aromatic structure may enable deeper engagement with the catalytic cleft, forming multiple stabilizing interactions. Interestingly, the enzyme DHODH from *P. falciparum*, a target in antimalarial therapy, was best inhibited by reticuline (−8.3), with linearisine (−8.2) following closely, while the co-crystallized ligand DZB scored −8.4 kcal/mol (Table 2). These values indicate that certain alkaloids can closely mimic, or even rival, the binding efficiency of synthetic drugs in terms of predicted binding energy

The comparative chart underscores the fact that while the co-crystallized ligands generally perform well, several alkaloids approach or surpass their affinity, particularly in *Leishmania* and *Trypanosoma* targets. Notably, norsalutaridine and reticuline consistently achieve strong binding across all selected enzymes. This cross-target activity, combined with their natural origin and traditional medicinal use, positions these compounds as valuable leads for further optimization. Homolinearisine and linearisine also exhibit moderate but consistent affinity, suggesting a favorable chemical scaffold.

Likewise, García Díaz et al. [6] reported significant in vitro antiparasitic activity for *C. linearis* extracts, attributing part of this efficacy to the presence of reticuline, glaucine, and related alkaloids. Together, these results support the computational predictions and reinforce the pharmacological relevance of these natural compounds as promising scaffolds for future drug development.

The docking score results provided an initial indication of the binding affinity of the alkaloids toward each target enzyme; however, a deeper understanding was achieved through the analysis of molecular interactions within the active sites. Compounds with the most favorable docking scores, such as reticuline and norsalutaridine, consistently established key stabilizing interactions, such as hydrogen bonds, hydrophobic contacts, and π–π stacking, mirroring or surpassing those formed by the co-crystallized ligands. These interactions not only validate the docking predictions but also highlight the structural features responsible for their strong enzyme binding.

### 2.1. Interaction T. cruzi 14α-Demethylase (Cyp51) with Reticuline, Linearisine, and Norsalutari-Dine

**Figure 2:**
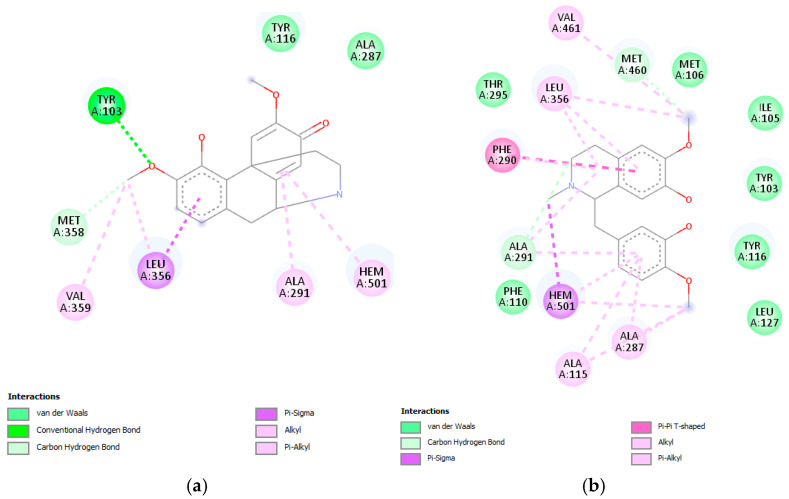
Docked structure of alkaloids norsalutaridine (**a**) and reticuline (**b**) in *T. cruzi* Cyp51 target, describing the amino acid residues of the catalytic site involved in the complex stabilization.

Sterol 14α-demethylase is a cytochrome P450 monooxygenase (Cyp51) that catalyzes oxidative removal of the 14α-methyl group from sterols and is an essential enzyme in sterol biosynthesis. According to Lepesheva et al. in 2010 [13], the active site involves the amino acids Tyr103, Met106, Phe110, Tyr116, Leu127, Ala297, Phe290, Ala291, Thr295, Leu356, Leu357, Met358, Met360, Val461, Met460, and Ala287. The interaction of alkaloids with *T. cruzi* Cyp51 revealed that several metabolites from *C. linearis* displayed docking scores very close to or comparable with the co-crystallized ligand NEE (−9.1 kcal/mol). Notably, norsalutaridine (−8.8 kcal/mol) and reticuline (−8.3 kcal/mol) demonstrated strong affinities, forming multiple stabilizing interactions within the enzyme’s active site (Figure 2), slightly better than co-crystallized ligand TPF (−8.0 kcal/mol). From a structural point of view, these alkaloids maintain aromatic moieties and basic nitrogen atoms that enable π-stacking and hydrogen bonding interactions with key residues in the heme-containing active pocket, like standard azole-based inhibitors.

These interactions are supported by visualizations of docked poses, where both (norsalutaridine and reticuline) are stabilized by contacts with Met105, Tyr116, and the heme group. Literature from recent years supports the role of Cyp51 as a validated target for Chagas disease chemotherapy. For instance, azole derivatives like posaconazole and ravuconazole have shown nanomolar affinities in vitro and in vivo [14]. The binding energies of alkaloids in this study, although slightly less potent, demonstrate promising leads for natural product-based inhibitors. Furthermore, homolinearisine and wilsonirine also perform comparably to the reference compound, reinforcing the potential of these molecules in lead optimization pipelines.

**Figure 3:**
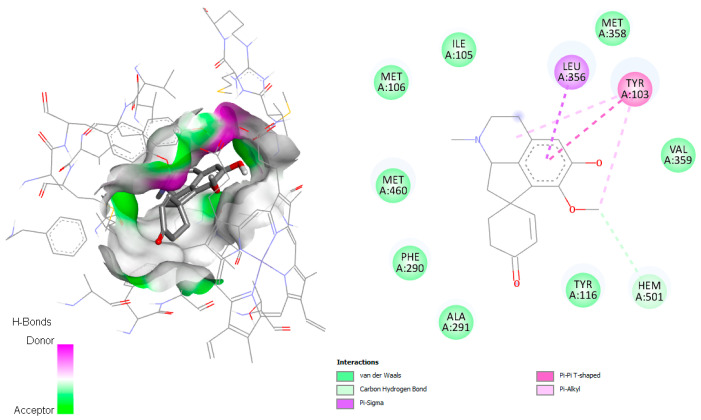
Docked structure of alkaloid linearisine in *T. cruzi* Cyp51 target, describing the amino acid residues of the catalytic site involved in the complex stabilization.

On the other hand, linearisine was found to interact with Leu356 by π–σ interaction and π–π T-shaped interaction with Tyr103, both involving the active site (Figure 3), in addition to many hydrophobic interactions involving different amino acid residues, including Met358, Val359, Ile105, Met106, Met460, Phe290, Ala291, and Ty116 present in the active site.

Co-crystallized ligand NEE was found to establish π–π interactions with Tyr116 and Phe290 in *T.cruzi* Cyp51 and demonstrated a π-σ interaction with Leu127. Moreover, for this complex, alkyl interactions with the amino acid residues Hem501, Ala287, Tyr103, Leu356, Ala115, Ala291, and Val461 were observed (Figure S1). Co-crystallized ligand NEE demonstrated similar interaction as norlutaridine, reticuline, and linearisine alkaloids; they share the same residues involved in the active site as Ty116, Met106, Met460, Phe290, Ala291, etc.

### 2.2. Interaction T. cruzi DHFR-TS with Laudanidine and Reticuline

Dihydrofolate reductase (DHFR) is a global enzyme that has been a well-known and effective target in medicinal chemistry not only due to its anticancer, antibacterial, and antimalarial treatments, but also due to its central role in cellular metabolism and DNA synthesis in *T. cruzi* [15].

**Figure 4:**
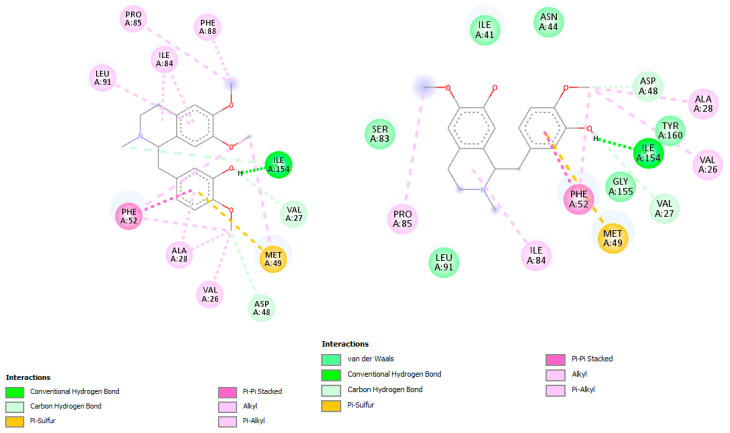
Docked structure of alkaloid laudanidine and reticuline in *T. cruzi* DHFR-TS target, describing the amino acid residues of the catalytic site involved in the complex stabilization.

**Figure 5:**
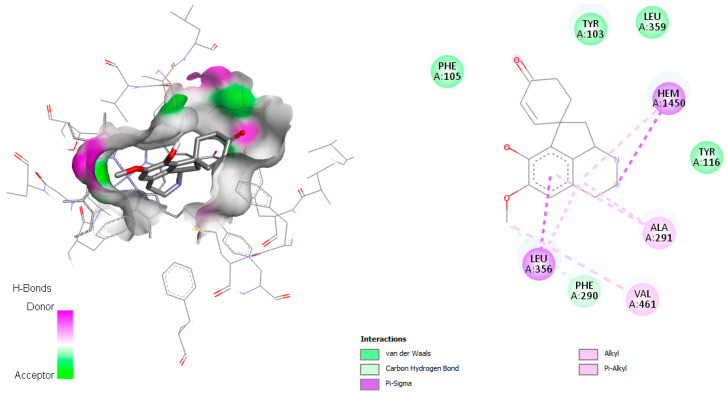
Docked structure of alkaloid jacularine in *T. brucei* Cyp51 target, describing the amino acid residues of the catalytic site involved in the complex stabilization.

In docking simulations against dihydrofolate reductase-thymidylate synthase (DHFR-TS), several *C. linearis* alkaloids outperformed the co-crystallized ligand 2CY. The top scorers, laudanidine and reticuline, exhibited stronger predicted binding affinities than the reference, suggesting a more stable enzyme-inhibitor complex. Structural docking analysis (Figure 4) shows these compounds fitting tightly into the folate-binding pocket, engaging in hydrogen bonds and hydrophobic interactions with active site residues such as Phe52, Phe-88, Asp48, Ala28, Gly156, Gly157, and Tyr16. Other interactions with hydrophobic contact and weak bonds involved Ile41, Ile35, Lys79, Tyr160, Thr80, Pro85, and Ile84 (Figure 5). These findings align with recent drug discovery efforts [16]. For example, the repurposing of antifolates for trypanosomatids has regained interest due to cross-species conserved domains in DHFR [17]. Some studies have also reported activity of natural hemisynthetic alkaloids against DHFR in *Plasmodium* species, which supports the plausibility of these findings [18].

The *T. cruzi* DHFR enzymes differ from other enzymes in that they are found as a bifunctional homodimeric protein with the thymidylate synthase (TS) domain located at the C-terminal. The overall structure of DHFR-TS is very flexible, and the DHFR domain is at the N-terminus and is separated from the TS domain (C-terminus) by a peptide linker that varies in size. In most organisms, DHFR and TS exist as separate monofunctional enzymes. According to Beltran-Hortelano et al. in 2022 [15], DHFR-TS has three regions with different characteristics: region I is composed of alkyl and aromatic side chains, region II has a hydrophilic character formed by hydrophilic amino acids and carbonyl (C=O) or amine (NH) groups of the peptide bonds, and region III has an arginine capable of bridging hydrogen.

### 2.3. Interaction T. brucei 14α-Demethylase (Cyp51) with Jacularine

According to Choi et al., 2013 [19], the *T. brucei* 14α-demethylase (Cyp51) has 85% sequence identity to *T. cruzi* 14α-demethylase (Cyp51). Many of the first-tier active-site residues in these two parasite Cyp51 enzymes are identical, except for amino acid residue Phe105, which is isoleucine in the *T. cruzi* counterpart.

The Cyp51-jacularine complex demonstrated π–σ with Leu356 residue and heme group (Figure 5). Other favorable interactions of different types were formed, as van der Waals and π–alkyl interactions with several residues (Phe105, Tyr103, Leu359, Tyr116, Phe290, Ala291, and Val461) involved in the active site [13].

### 2.4. Interaction T. brucei Adenosine Kinase (ADkinase) with Reticuline

Adenosine kinase (ADkinase) catalyzes the phosphorylation of adenosine-to-adenosine monophosphate (AMP) in presence of Mg^2+^ and utilizing ATP as the preferred phosphoryl donor. The corresponding adenosine kinase of *T. b. rhodesiense* (TbrAK) is an enzyme that functions as a monomer, a common characteristic of other adenosine kinases. In general, the sequence identity among adenosine kinases of different species is moderate (17% to 40%); however, their structures are remarkably similar. The catalytic site of ADkinase involved Asn13, Ile38, Ser64, Phe169, Thr172, Asp299 (adenosine binding pocket), R132, N222, T264, G296, A297 (pentaphosphate moiety), and Arg265, Glu268, Thr270 (ATP binding pocket).

**Figure 6:**
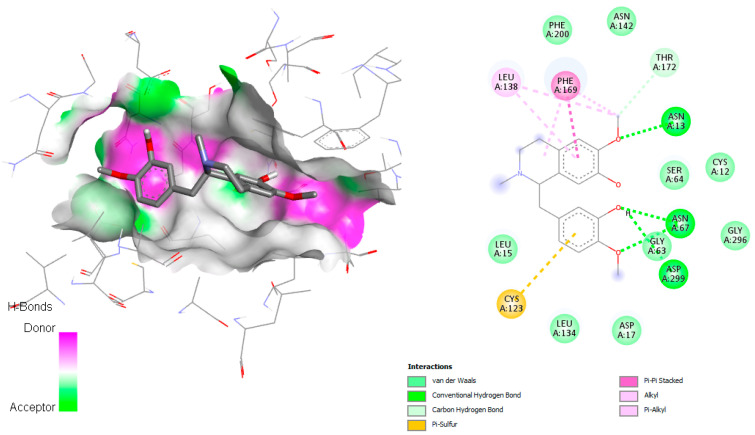
Docked structure of alkaloid reticuline in *T. brucei* ADkinase target, describing the amino acid residues of the catalytic site involved in the complex stabilization.

The docking simulation of reticuline in *T. brucei* ADkinase target exhibited an H-bonding interaction with Asn13, Asn67, and Asp299 through the oxygen atom of the hydroxyl group present in reticuline involved in the adenosine binding pocket reported by Kuettel et al., 2011 [20] (Figure 6). Two interactions with Phe169 (π–π stacked) and Cys123 (π–sulfur) were observed, as well as hydrophobic interactions, mainly van der Waals interactions (Phe200, Asn142, Thr172, Cy12, Ser64, Gly296, Gly63, Asp17, Leu134, Leu15), which are involved in the active site.

The docking interaction of the co-crystallized ligand KRM demonstrated π–σ interaction with Leu138, π–π interaction with Phe200, Phe204, and Phe169 (Figure S2). On the other hand, different hydrophobic interactions in the active site were observed, mainly van der Waals and alkyl interactions with the amino acids Leu15, Leu39, Ser64, Gly63, Cys12, Asn13, Asn142, Thr172, and Phe205. The reticuline and co-crystallized ligand KRM share some residues, Phe169, Leu138, and Phe200, involved in the active site, according to Kuettel et al., 2011 [20].

The docking simulations against *T. brucei* adenosine kinase (ADKinase) revealed that reticuline had the most favorable binding among the alkaloids, although it did not outperform the co-crystallized ligand KRM (–8.8 kcal/mol). The active site interactions shown in Figure 6 demonstrate hydrogen bonding and van der Waals contacts, particularly with key residues like Asp299 and Phe169 [21,22]. Despite slightly lower docking scores than the control, these metabolites may still offer biological relevance, especially considering the known flexibility and plasticity of the ADKinase pocket. Previous studies have shown that selective inhibitors for trypanosomal ADKinase can block purine salvage, critical for parasite survival [21]. Some alkaloids, such as crotonosine and linearisine, approach −7.8 kcal/mol, suggesting that chemical optimization (e.g., adding hydrogen bond donors or planar groups) could enhance binding affinity further. Moreover, these molecules may offer structural diversity compared to existing ADKinase inhibitors like tubercidin analogs [22].

### 2.5. Interaction L. infantum 14α-Demethylase (Cyp51) with Reticuline and Wilsonirine

**Figure 7:**
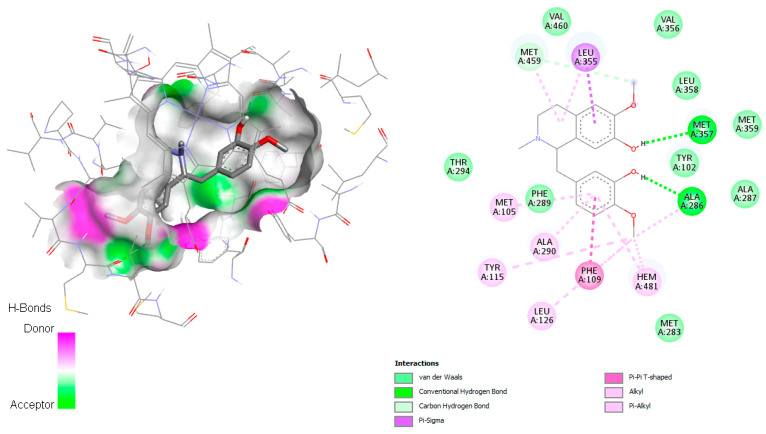
Docked structure of alkaloid reticuline in *L. infantum* Cyp51 target, describing the amino acid residues of the catalytic site involved in the complex stabilization.

Both compounds established key stabilizing interactions within the catalytic site of the enzyme (Figure 6 and Figure 7), including hydrogen bonds and π–π stacking with residues such as His99, Tyr116, and the heme prosthetic group, which plays a critical role in the enzyme’s function. These interactions suggest that the aromatic moieties of the alkaloids are well-oriented within the active site, mimicking the binding mode of the co-crystallized ligand.

The structural comparison with the native ligand revealed that while the co-crystallized TPF (Figure S3) primarily stabilizes through coordination with the heme iron and polar interactions with residues in the active channel, reticuline and norsalutaridine extend further into the hydrophobic pocket, enabling additional van der Waals contacts that may enhance binding stability. This extended coverage could confer greater inhibition potential, especially considering the structural rigidity and electron-rich features of their benzylisoquinoline scaffolds. Importantly, the docking poses retained the orientation required for interaction with the central heme group, a characteristic essential for successful Cyp51 inhibition.

Recent literature continues to underscore the potential of isoquinoline alkaloids as inhibitors of sterol biosynthesis in *Leishmania*. A study by Bessa et al. (2024) identified a range of isoquinoline and indole alkaloids from Amaryllidaceae showing potent *in vitro* activity against *L. infantum* [23], with molecular docking indicating strong binding to the parasite’s sterol 14α-demethylase (CYP51) and key π–π interactions within the heme pocket [24]. Additionally, research by Tanwar et al. (2024) [25] highlighted that antifungal azoles and azasterols, known to engage both π–π stacking and iron coordination, effectively inhibited *L. amazonensis* sterol biosynthesis, disrupting membrane integrity and resulting in parasite death [23]. These findings reinforce the concept that alkaloids like reticuline and norsalutaridine, with aromatic scaffolds capable of similar interactions, represent promising leads for Cyp51-focused drug development against *Leishmania*. Thus, the alkaloids identified in this study, particularly reticuline and norsalutaridine, may serve as promising scaffolds for further optimization against *L. infantum* Cyp51.

**Figure 8:**
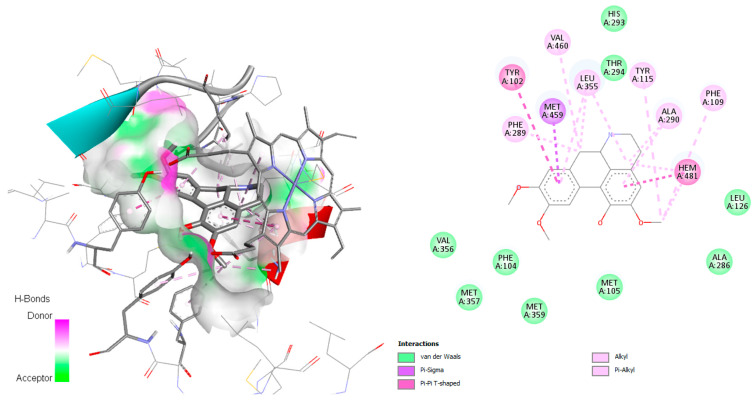
Docked structure of alkaloid wilsonirine in *L. infantum* Cyp51 target, describing the amino acid residues of the catalytic site involved in the complex stabilization.

According to Hargrove et al. in 2011 [26], the active site involved the amino acids Leu355, Met357, Leu358, Met459, Val212, Phe104, Met105, Tyr115, Ala114, Ala286, Phe109, Gly282, Met283, Phe289, Leu129, Ala290, and Val460. Reticuline was found to establish hydrogen bonds with Met357 and Ala286 (Figure 7), both involved in the active site. Additionally, for this complex, π–π T-shaped with Phe109, π–σ interaction with Leu355, and Van der Waals and alkyl interactions with the amino acid residues Hem371, Leu358, Val 460, Val356, Leu358, Met359, Tyr102, Ala287, Met283, Ala290, Met205, Tyr115, Phe289, and Leu126 were observed. Wilsonirine demonstrated π–π T-shaped interaction with Hem481 and Tyr102 (Figure 8), besides hydrophobic interaction in the active site, van der Waals and alkyl interactions were observed mainly with Val460, Phe109, Val356, Leu355, Ala286, Leu358, Met359, Tyr102, Ala287, Met283, Ala290, Met205, Tyr115, Met357, Phe289, and Leu126, all involved in the active site [26].

The co-crystallized ligand TPF was found to establish halogen (fluorine) interaction with Ala290 and Hem481 (Figure S3), demonstrate π–σ interaction with Leu355 and Met459, van der Waals and alkyl interaction with Phe104, Met105, Phe289, Phe109, Tyr115, Tyr102, Leu126, Thr294, and Ala286. Reticuline and wilsonirine share some residues with the co-crystallized ligand, as Ala290, Phe109, Hem481.

In the case of *L. infantum*, norsalutaridine again emerged as the top-performing alkaloid with a docking score of −9.3 kcal/mol, surpassing the co-crystallized ligand TPF. This result indicates a potential dual inhibitory profile against *T. cruzi* and *Leishmania* Cyp51 enzymes. Docked conformations (Figure 6, Figure 7 and Figure S3) show consistent interaction networks, with hydrophobic contacts and hydrogen bonding contributing to binding stability. These findings are notable given the limited number of approved treatments for visceral leishmaniasis and the known limitations of azole-based drugs, including resistance and toxicity [26]. The high binding affinities and favorable interactions of wilsonirine and salutaridine support their potential as scaffolds for novel CYP51 inhibitors.

Recent docking studies by Bessa et al. (2024) [23] revealed that isoquinoline alkaloids isolated from *Hippeastrum aulicum* exhibit strong binding affinity toward sterol 14α-demethylase (Cyp51) in *L. infantum*, primarily through π–π stacking interactions and hydrogen bonding within the heme-binding site. However, their predicted binding energies were generally less favorable compared to those of the *C. linearis* alkaloids evaluated in the present study. These findings support the hypothesis that *C. linearis* metabolites, particularly reticuline and norsalutaridine, may constitute a distinct subclass of alkaloids with superior structural compatibility and binding strength against Cyp51 enzymes.

### 2.6. Interaction L. major Pteridine Reductase 1 (PTR1) with Reticuline and Wilsonirine

**Figure 9:**
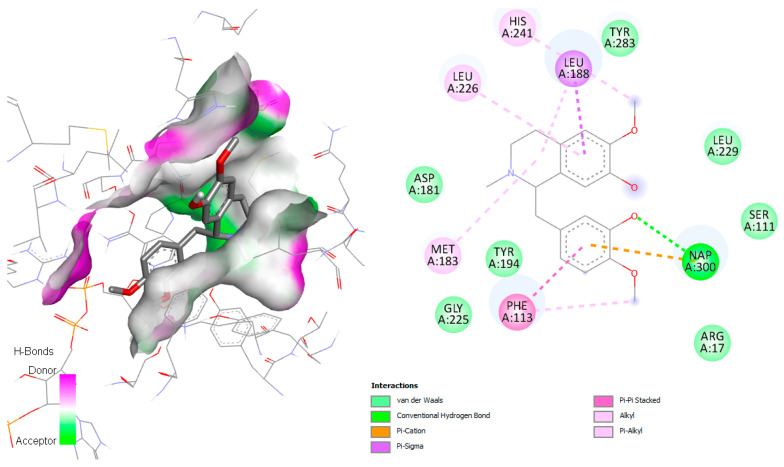
Docked structure of alkaloid reticuline in *L. major* PTR1 target, describing the amino acid residues of the catalytic site involved in the complex stabilization.

**Figure 10:**
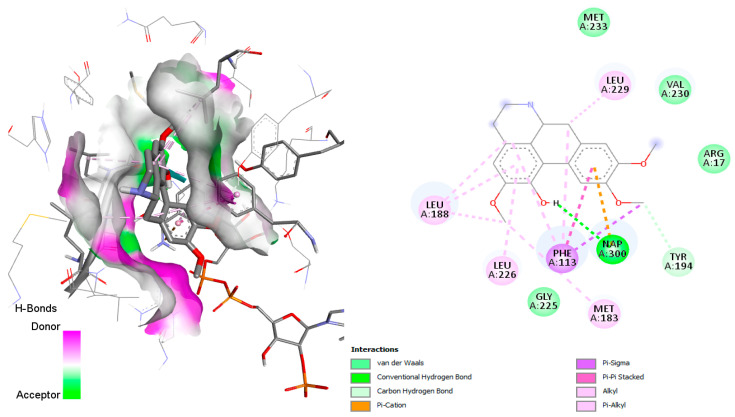
Docked structure of alkaloid wilsonirine in *L. major* PTR1 target, describing the amino acid residues of the catalytic site involved in the complex stabilization.

The pteridine reductase 1 (PTR1) enzyme plays an adaptive role in antifolate resistance, making it a validated drug target in *Leishmania* species. In this study, several *C. linearis* alkaloids demonstrated superior binding to PTR1 compared to the reference compound. Notably, laudanosine and reticuline yielded docking scores of −8.8 and −8.7 kcal/mol, respectively, which indicates higher predicted affinity. Figure 9 and Figure 10 show that these alkaloids form extensive hydrogen bonds and π–π stacking interactions with conserved residues such as Ser111 and Phe113, involved in substrate recognition. This is consistent with known interactions reported for methotrexate analogs and benzoquinolines [27], which also demonstrated strong binding to *Leishmania* PTR1 through hydrogen bonds with Ser111 and π–π stacking with Phe113. Singh et al. [28] demonstrated that these interactions are critical for inhibitor efficacy, as they stabilize the ligand within the enzyme’s active site. The similar binding patterns observed in our study with *C. linearis* alkaloids, particularly laudanosine and reticuline, suggest that these natural compounds may exploit the same mechanistic pathways as synthetic inhibitors, further validating PTR1’s conserved binding features as a target for antifolate resistance. Given that PTR1 inhibitors are scarce in the clinical pipeline, these results highlight the potential of natural isoquinoline scaffolds to serve as new leads. Moreover, wilsonirine also demonstrated strong binding (−8.9), warranting further evaluation through in vitro enzyme inhibition studies.

### 2.7. Interaction P. falciparum Dihydroorotate Dehydrogenase (DHODH) with Jacularine, Reticuline, and Linearisine

**Figure 11:**
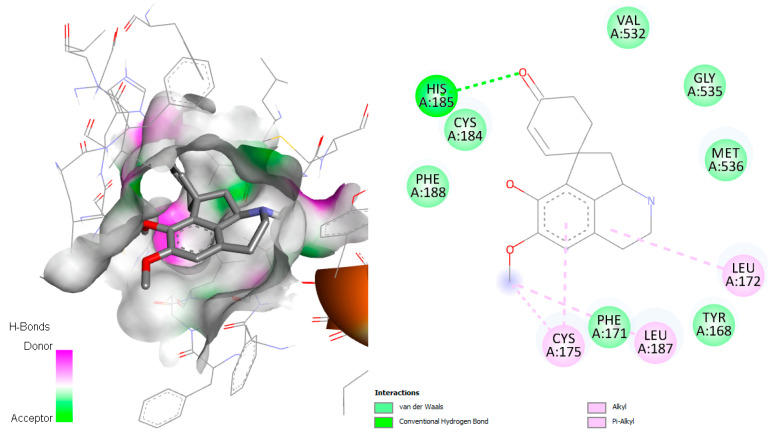
Docked structure of alkaloid jacularine in *P. falciparum* DHODH target, describing the amino acid residues of the catalytic site involved in the complex stabilization.

The target of *P. falciparum* dihydroorotate dehydrogenase (PfDHODH) is an enzyme involved in the catalysis of the fourth step in de novo pyrimidine biosynthesis. The de novo pyrimidine biosynthesis pathway is crucial to the survival of the parasite. Unlike human cells, which can utilize the salvage pathway for pyrimidine acquisition,

*Plasmodium* species can only access de novo-synthesized pyrimidines. According to Pippione et al., 2019 [29], the active site of this enzyme contains numerous amino acid residues, including Tyr168, Phe171, Leu172, Ile176, Leu187, Phe188, Tyr528, Phe227, Phe188, Cys175, Leu531, Ile263, His185, Ile272, Arg265, and Met536. The *P. falciparum*_DHODH-jacularine complex demonstrated hydrogen with His185, similar to the result obtained by Pippione et al. [29]. In addition, different hydrophobic interactions in the active site were observed, mainly van der Waals and alkyl interactions with the amino acids Val532, Cys184, Phe188, Gly535, Met536, Leu172, Tyr168, Leu187, Phe171, and Cys175 (Figure 11).

**Figure 12:**
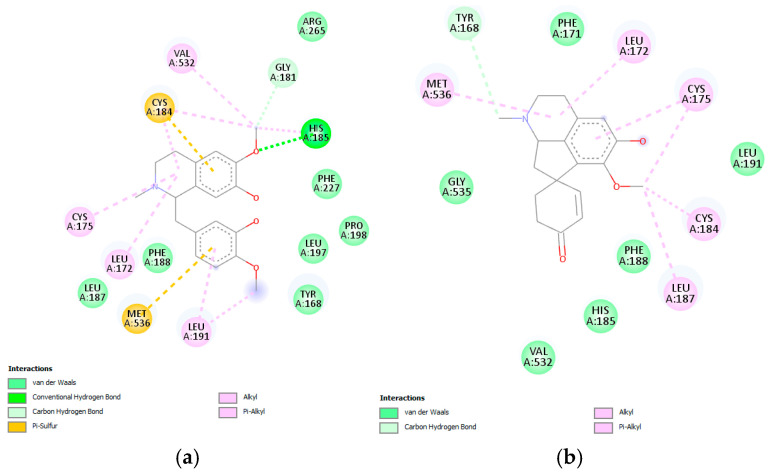
Docked structure of alkaloids reticuline (**a**) and linearisine (**b**) in *P. falciparum* DHODH target, describing the amino acid residues of the catalytic site involved in the complex stabilization.

Reticuline and linearisine demonstrated similar interactions like jacularine (Figure 12); however, only the reticuline was found to establish hydrogen bonds with His185, and π–sulfur interactions with Cys184 and Met536 were observed (Figure 12a). On the other hand, linearisine demonstrated no hydrogen bond interactions; however, it exhibits several hydrophobic interactions, as van der Waals interactions with the Gly535, Phe171, Leu191, Phe188, His185, and Val532, alkyl and π–alkyl interactions with Met536, Leu172, Cys175, Cys184, Leu187 residues (Figure 12b). Reticuline and linearisine share several amino acids—Phe171, Leu172, Leu187, Phe188, Phe188, and Met536—involved in the active site of the *P. falciparum* DHODH enzyme.

Inhibitors of *P. falciparum* DHODH are effective antimalarial agents due to their role in pyrimidine biosynthesis. The best-performing alkaloids in this study, reticuline and jacularine, demonstrated binding energies very close to the reference compound DZB (−8.4 kcal/mol), as observed in Figure 10 and Figure 11. These compounds aligned well with the ubiquinone-binding pocket of *Pf*DHODH, forming key interactions with residues such as Arg265, Tyr528, and Phe188. These findings align with studies on triazolopyrimidine-based compounds (DSM265) analogs [30], which report similar hydrophobic and π-stacking contacts. Moreover, linearisine and homolinearisine exhibited good binding (−8.1 to −8.2 kcal/mol), quite similar to the value achieved with the co-crystallized ligand (−8.4 kcal/mol), suggesting that multiple alkaloids share a common pharmacophore motif.

The comparative analysis of docking scores across parasite enzymes and their human homologs revealed a heterogeneous pattern of selectivity among the tested alkaloids (Table S3a,b). Compounds such as reticuline, laudanidine, and linearisine consistently displayed positive ΔScore values in several targets, particularly against *Trypanosoma cruzi* cruzain, *Leishmania* FPPS, and *Plasmodium falciparum* DHODH. Positive ΔScore values suggest stronger predicted binding to the parasite enzyme compared to the human counterpart, highlighting these molecules as potential candidates with favorable selectivity. Conversely, several compounds, including corydine and glaucine, presented predominantly negative ΔScore values in multiple comparisons, reflecting either non-selective binding or stronger interactions with human homologs, thus raising potential concerns about off-target effects.

In *Trypanosoma* targets (Table S3a), the selectivity profile was variable. While compounds like reticuline and laudanidine exhibited promising selectivity against cruzain and DHFR-TS, molecules such as pronuciferine and jacularine showed negative ΔScore values for the same enzymes, suggesting stronger interactions with human cathepsins and DHFS. Interestingly, n-methylhernovine and litseglutine B displayed extreme variations, with strongly positive ΔScore values against *T. cruzi* cruzain but markedly negative ones against other targets, emphasizing their context-dependent selectivity. These findings underscore the complexity of achieving broad parasite selectivity within this alkaloid series.

For *Leishmania* enzymes (Table S3b), particularly FPPS, TryR, and cyp51, the results highlighted reticuline, linearisine, and homolinearisine as molecules with favorable parasite-biased binding, as reflected in positive ΔScore values. However, a recurring observation was the loss of selectivity in *L. infantum* TryR and *L. mexicana* ArgI, where most compounds displayed strongly negative ΔScore values, showing preferential affinity toward the human homologs. This suggests that enzyme conservation between parasite and human homologs may limit the therapeutic window for certain targets. In the case of *P. falciparum*, compounds like cularine, wilsonirine, and crotonosine exhibited notable selectivity toward DHODH and falcipain, consistent with previous studies finding these enzymes as exploitable selective targets in antimalarial drug discovery.

Overall, the ΔScore analysis shows that while several alkaloids, including reticuline, laudanidine, and linearisine, show promising selectivity for parasite enzymes, none of the tested molecules achieved uniform selectivity across all species and targets. The observed variability highlights the necessity of a target-specific approach, where alkaloid scaffolds may be customized for distinct parasite enzymes rather than seeking broad-spectrum selectivity. Future optimization should focus on compounds with consistently positive ΔScore values while also integrating ADMET predictions to mitigate the risks of off-target interactions with human homologs.

### 2.8. Overall ADMET Profile and Prioritization of Alkaloids Candidates

In silico ADMET evaluation of the *C. linearis* derived alkaloids (Table S4) provided critical insights into their pharmacokinetic behavior and potential safety liabilities, complementing the molecular docking analysis against neglected tropical disease targets. The objective of the present study was to identify candidate molecules with favorable bioavailability, minimal toxicity risks, and optimal drug-like properties. This was achieved by assessing absorption, distribution, metabolism, excretion, and toxicity parameters. The integration of these predictive data with binding affinity profiles offers a more comprehensive understanding of each alkaloid’s therapeutic potential, thereby guiding the prioritization of compounds for future in vitro and in vivo validation.

Across the 18 alkaloids, physicochemical properties cluster tightly within an “orally developable” window: MW 283–357 g/mol (median ≈ 327), XLogP3 1.48–3.67 (median ≈ 2.55), and TPSA 38.8–71.0 Å2 (median ≈ 54.9). It is notable that none of the compounds under examination violate the Lipinski, Veber, Egan, or Muegge filters, thereby aligning with established correlates of good oral exposure (≤5 HBD, ≤10 HBA, MW ≤ 500, cLogP ≤ 5; ≤10 rotatable bonds and TPSA ≤ 140 Ų). The findings of this study align with the hypothesis that consistently high GI absorption predictions further support oral suitability. All molecules are designated as BBB-permeant, a classification that is consistent with their low TPSA (<90 Ų) and moderate lipophilicity. These physicochemical characteristics are well-documented as being associated with CNS access. These observations are consistent with the established principles of classical oral drug-likeness literature (Lipinski; Veber) [31] and with SwissADME’s BOILED-Egg rationale [32], which establishes a correlation between lipophilicity/polarity and GI and brain permeation.

However, predictions concerning transport and metabolism introduce a degree of complexity to this interpretation. The P-gp [33] substrate status was found to be positive for 15 out of 18 compounds, with only laudanosine, laudanidine, and pronuciferine predicted to be non-substrates. This suggests the possibility of efflux-limited brain exposure despite passive BBB permeability [34]. This finding aligns with the established role of P-gp as a gatekeeper at the BBB, as well as with clinical imaging and PK evidence demonstrating reduced unbound brain partitioning for P-gp substrates. With regard to the subject of metabolism, CYP2D6 inhibition (16/18) and CYP3A4 inhibition (11/18) have been observed, indicating the potential for drug–drug interactions (DDIs) with substrates of these polymorphic/high-clearance pathways. In contrast, fewer compounds have been found to inhibit CYP2C9 (2/18) or CYP2C19 (1/18). Such inhibition patterns are consistent with the high clinical interaction liability of 2D6/3A4, as highlighted in recent reviews of CYP-mediated DDIs [35].

From a medicinal chemistry quality perspective, the set is considered to be clean, as it does not contain any PAINS (Potential Adverse Drug Reactions) [36] or Brenk structural alerts. This characteristic supports the notion that there is a reduced risk of assay interference and toxicity, and it is well-suited for screening purposes. Lead-likeness is also strong (16/18 with zero violations), with only glaucine (1 violation) and laudanosine (2 violations) flagged. This is useful for prioritization if fragment/lead-like progression is desired. The presence of a bioavailability score (typically 0.55 in SwissADME for Ro5-compliant, nonpolar zwitterions) complements the absorption predictions. Collectively, these results mirror widely utilized quality filters (PAINS/Brenk) and SwissADME guidance for early triage [37].

## 3. Materials and Methods

A series of computational experiments were conducted using the High-Performance Computing (HPC-UO) cluster of the Oriente University, accessed on 8 May 2024 (https://portal.uo.hpc.cu/website/, accessed on 8 May 2024). Molecular docking simulations were performed with AutoDock Vina version 1.2 [38], a widely employed program for predicting protein–ligand interactions with improved accuracy and efficiency. The configuration of the system was such that it guaranteed adequate computational capability and dependability for the substantial docking calculations that were necessitated by the study.

### 3.1. Preparation of the Ligands and Target Structures

#### 3.1.1. Ligands

The structures of alkaloids were drawn with correct stereochemistry using Chemdraw and Chem3D (Version 20.1, PerkinElmer Informatics, Waltham, MA, USA) [39], nonpolar H atoms were merged onto all ligands. The structures were geometry optimized using the MMFF94 force field.

#### 3.1.2. Proteins

Protein-ligand docking studies were carried out based on the crystal structures of verified different neglected diseases protein drug targets: *T. cruzi* cruzain (PDB 3IUT) [40], *T. cruzi* sterol 14α-demethylase Cyp51 (PDB 4H6O) [41], *T. cruzi* dihydrofolate reductase-thymidylate synthase DHFR-TS (PDB 3IRO) [42], *T. cruzi* triosephosphate isomerase TIM (PDB 1SUX) [43], *T. brucei* sterol 14α-demethylase Cyp51 (PDB 4BJK) [19], *T. brucei* rhodesain (PDB 2P7U) [44], *T. brucei* enolase (PDB 2PTY) [45], *T. brucei* adenosine kinase ADNkinase (PDB 2XTB) [20], *L. infantum* sterol 14α-demethylase Cyp51 (PDB 3L4D) [26], *L. infantum* trypanothione reductase TryR (PDB 2JK6) [46], *L. mexicana* arginase I ArgI (PDB 4ITY) [47], *L. major* nucleoside diphosphate kinase b NDKb (PDB 3NGU) [48], *L. major* farnesyl diphosphate synthase FPPS (PDB 4JZX) [49], *L. major* pteridine reductase 1 PTR1 (PDB 3H4V) [50], *P. falciparum* plasmepsin2 (PDB 2BJU) [51], *P. falciparum* dihydroorotate dehydrogenase DHODH (PDB 6I55) [29] and *P. falciparum* falcipain-3 (PDB 3BWK) [44].

The different protein structures contain identical domains; for these proteins, we used chain A for each protein. Previous to docking, the solvents and co-crystallized ligands from the structures were removed. Using AutodockTools 1.5.6 (ADT) [52], Kollman united atom charges, solvation parameters, and polar hydrogens were added to the protein for the preparation of the enzyme in the docking simulations. Since the ligands are not peptides, Gasteiger charges were assigned, and nonpolar hydrogens were merged.

The grid box was centered around the region of interest (catalytic site) on the position of the co-crystallized ligand in the macromolecule (Table S1), and the grid box size was 24 × 24 × 24. In the case of ArgI, TryR, and NDKb target structures, no inhibitors were present, and the known antileishmanial drug pentamidine, taken from DrugBank [53], was used.

### 3.2. Molecular Docking Parameters and Validation

All the docking calculations were performed applying recently introduced by Scripps Research Institute AutoDock Vina version 1.2 [38]. To validate the molecular docking protocol employed in this study, the co-crystallized ligands of each target enzyme were redocked into their respective binding sites, and root mean square deviation (RMSD) was calculated. Docking simulations were conducted using AutoDock Vina (version 1.2), with the exhaustiveness parameter set to 10 to ensure sufficient sampling of the conformational space, and the number of output binding modes constrained to 10 for each ligand-receptor complex. According to the literature, an RMSD ≤ 2.0 Å in re-docking is generally accepted as an indication of reliable reproduction of the experimental pose, although the success rate may vary depending on the protein and the size or flexibility of the ligand [54].

The binding affinities and interaction profiles obtained from the redocking simulations were compared to those of the original co-crystal structures. This validation step ensured that the docking approach could reliably reproduce the key interactions and binding modes observed experimentally, thereby providing confidence in the subsequent docking results of the test compounds (see Table 1 and Table 2).

To visually analyze the ligand–receptor interactions, Discovery Studio Visualizer version 21.1 (BIOVIA, Dassault Systems) [55] was employed. This software enabled the generation of both 2D and 3D interaction diagrams, facilitating the detailed interpretation of binding modes within the active sites of the target enzymes. The 2D diagrams allowed for the clear identification of hydrogen bonds, hydrophobic contacts, and other key non-covalent interactions, while the 3D representations provided spatial context to assess the fitting of the ligands in the binding pockets. The use of Discovery Studio significantly enhanced the clarity and accuracy of interaction analyzes, highlighting its utility as a robust tool in structure-based drug design workflows.

Docking-based selectivity analysis was performed by comparing the predicted binding energies of each ligand against the parasite target and its closest human ortholog. Molecular docking simulations were carried out using AutoDock Vina, and the binding energies (Δ*G*, kcal·mol^−1^) were extracted from the docking output. Docking scores were expressed as predicted binding energies in kcal·mol^−1^ (more negative = stronger predicted binding). For each compound, a computed ΔScore [56] was calculated as follows:

ΔScore = Score_human − Score_parasite

where Score_human and Score_parasite are the representative docking scores against the human homolog and the parasite target, respectively. Docking was performed for each receptor using identical protocols and scoring functions; for each ligand, the best-ranked pose (lowest energy) and the mean of the top three poses were recorded, and both were used to compute ΔScore (reporting the mean reduces sensitivity to pose outliers). By this sign convention, a positive ΔScore indicates preferential binding to the parasite (i.e., Score_parasite is more negative than Score_human). The primary filters used were as follows: (i) Score_parasite ≤ −6.0 kcal/mol to indicate a promising absolute affinity and (ii) ΔScore ≥ 1.0 kcal/mol as a minimal selectivity window; compounds with ΔScore ≥ 2.0 kcal/mol were prioritized as strongly selective hits. Large positive values (i.e., when the human score is less negative than the parasite score) show greater selectivity towards the parasite (because the ligand binds more strongly to the parasite than to humans). Positive ΔScore values show a preferential affinity toward the parasite protein, while negative values suggest higher affinity for the human counterpart. Ligands were ranked based on their ΔScore values, and compounds with more favorable parasite selectivity (i.e., significantly positive ΔScore) were prioritized for further analysis.

In several cases (Cyp51, FPPS, DHODH, FPPS, enolase, TIM, ADK, ARG1, FPPS, NME1) (Table S2), a direct human homolog with available crystallographic structures is present, facilitating in silico studies of selectivity (comparing active sites, superimposing structures). In certain cases, the parasitic enzyme lacks a direct human equivalent (for instance, PTR1 and TryR; the latter has no exact human substrate analog). In such cases, it is advisable to note in the manuscript that there is no direct human homolog and to provide a justification for why that target is attractive (e.g., parasitic specificity, lower risk of toxicity if selectivity is maintained). For cysteine/aspartic proteases (cruzain, rhodesain, falcipain, plasmepsins), the human papain/pepsin/cathepsin family contains the relevant homologues (cathepsin L/D). Consequently, structural comparisons with these human cathepsins are imperative to estimate off-target risk.

### 3.3. ADMET Prediction Methodology

ADMET predictions were performed using the SwissADME online platform [37], which provides a comprehensive in silico evaluation of pharmacokinetic, drug-likeness, and medicinal chemistry properties. The chemical structures of the alkaloids isolated from *Croton linearis* were prepared in canonical SMILES format and individually submitted to SwissADME for analysis. Key parameters assessed included molecular weight (MW), topological polar surface area (TPSA), consensus LogP, gastrointestinal (GI) absorption, blood–brain barrier (BBB) permeability, cytochrome P450 (CYP) isoform inhibition, Brenk alerts, and lead-likeness violations. The prediction outputs were subsequently compiled for comparative analysis, with special emphasis on pharmacokinetic suitability and medicinal chemistry friendliness in the context of potential antiprotozoal drug candidates.

## 4. Conclusions

Integrated docking and ADMET analyses of *C. linearis* Jacq alkaloids highlight their potential as versatile scaffolds in the discovery of drugs for neglected tropical diseases (NTDs). The docking simulations revealed potent multitarget inhibitory profiles, with metabolites such as reticuline, norsalutaridine, laudanosine, and jacularine exhibiting binding affinities comparable to or even greater than those of co-crystallized ligands against key protozoan enzymes, including Cyp51, DHFR-TS, and PTR1. Importantly, the calculated ΔScore values (Δ*G*_human − Δ*G*_parasite) consistently favored parasite enzymes, indicating a higher selectivity towards the protozoan targets over their human homologues. This trend strongly suggests that these alkaloids could exert specific antiparasitic activity through mechanisms consistent with the disruption of ergosterol biosynthesis and antifolate-like pathways.

In addition, the chemical diversity of alkaloids, one of the most widely studied and distributed classes of secondary metabolites, reinforces their potential as a source of novel therapeutic scaffolds. Taking into account present results, could be interesting to assay other known alkaloids against the studied parasite targets. The current findings suggest that the selective binding identification through ΔScore analysis could be extrapolated to other known alkaloids, thereby broadening the spectrum of candidates worth evaluating against the studied parasite targets. Nevertheless, different limitations of the present study require continuing further experiments. First, cross-docking experiments with X-ray structures of human orthologues should be prioritized, since most of the targeted parasite enzymes belong to conserved metabolic pathways. The identification of more favorable binding energies in parasites compared to humans, as supported by the present ΔScore results, would significantly strengthen the translational potential of these compounds for clinical applications. Second, in silico results should be corroborated *by* in vitro assays, preferably enzymatic and cellular models.

Complementary ADMET predictions reinforced the drug-like potential of the analyzed alkaloids, highlighting favorable oral bioavailability, high gastrointestinal absorption, strong drug-likeness scores, and the absence of major structural alerts. However, CYP450 inhibition, particularly of CYP2D6, remains a concern. Despite this limitation, several alkaloids (notably jacularine, linearicine, salutaridine, nor-salutaridine, and reticuline) showed balanced pharmacokinetic and toxicity profiles, making them the most promising candidates for further experimental validation. Overall, the combination of docking, ΔScore selectivity analysis, and ADMET profiling provides a rational basis for advancing selected alkaloids into in vitro and in vivo studies. Future research should validate the proposed molecular mechanisms using enzymatic and cellular models, while also focusing on metabolism, solubility optimization, and polypharmacological strategies to address the complexity of NTDs.

## Figures and Tables

**Table 1:** Docking score (Δ*G*_dock_) for the alkaloids from *Croton linearis* and the target enzymes chosen for *Trypanosoma* species.

*T. cruzi*					*T. brucei*			
Alkaloids	Cruzain (3IUT)	Cyp 51 (4H6O)	DHFR-TS (3IRO)	TIM (1SUX)	Cyp51 (4BJK)	Enolase (2PTY)	Rhodesain (2P7U)	ADkinase (2XTB)
Binding Energy (kcal/mol)								
Corydine	−6.7	−7.7	−7.5	−6.8	−8.5	−5.5	−6.3	−7.7
Cularine	−6.3	−7.9	−8.3	−7.5	−8.2	−5.7	−6.3	−7.8
Glaucine	−5.8	−7.2	−7.6	−6.6	−8.1	−5.5	−6.6	−7.1
Laudanosine	−6.6	−6.9	−7.8	−6.7	−8.3	−6.2	−6.6	−7.7
Laudanidine	−7.1	−8.1	−8.5	−6.4	−8.4	−6.1	−6.9	−7.9
Reticuline	−7.4	−8.3	−8.4	−7.2	−8.2	−6.2	−7.1	−8.1
Jacularine	−6.4	−8.0	−7.9	−8.2	−8.7	−6.2	−7.1	−7.2
Pronuciferine	−6.1	−7.5	−7.4	−8.2	−9.1	−5.5	−6.2	−7.1
Salutaridine	−6.2	−7.9	−7.8	−6.9	−9.0	−5.8	−6.7	−7.8
Norsalutaridine	−6.3	−8.8	−8.3	−6.7	−9.1	−6.1	−6.7	−7.6
Wilsonirine	−6.5	−7.7	−8.1	−8.0	−8.4	−5.8	−7.0	−7.3
Crotonosine	−6.8	−8.1	−7.8	−7.7	−8.5	−5.3	−6.9	−7.6
Homolinearisine	−6.9	−8.2	−8.0	−7.7	−8.3	−5.4	−6.8	−8.0
Linearisine	−6.9	−8.3	−8.1	−8.0	−8.2	−5.4	−7.0	−7.8
Hernovine	−5.8	−7.9	−7.0	−7.3	−8.4	−5.1	−6.0	−7.4
N-Methylhernovine	−5.9	−7.3	−7.1	−7.0	−8.2	−5.8	−6.2	−7.3
10-O-Methylhernovine	−6.1	−7.6	−7.1	−7.3	−8.4	−5.3	−6.1	−7.1
Litseglutine B	−5.9	−7.2	−6.9	−7.1	−8.2	−5.9	−6.1	−7.0
Co-crystallized ligand (RMSD, Å)	−6.4 (1.026)	−9.1 (1.202)	−7.7 (1.756)	−5.6 (1.639)	−11.9 (1.359)	−6.6 (1.700)	−6.3 (1.266)	−8.8 (1.580)

RMSD: Root mean square deviation.

**Table 2:** Docking score (Δ*G*_dock_) (kcal/mol) for the alkaloids from *Croton linearis* and the target enzymes chosen for *Leishmania* and *Plasmodium* species.

	*L. mexicana*	*L. infantum*		*L. major*			*P. falciparum*		
Alkaloids	ArgI (4ITY)	Cyp 51 (3L4D)	TryR (2JK6)	NDKb (3NGU)	FPPS (4JZX)	PTR1 (3H4V)	Plasmepsin2 (2BJU)	DHODH (6I55)	Falcipain3 (3BWK)
Binding Energy Dock (kcal/mol)									
Corydine	−6.6	−7.1	−4.8	−7.1	−6.9	−8.6	−6.7	−7.4	−7.3
Cularine	−6.8	−8.2	−5.3	−6.8	−7.2	−8.6	−6.7	−7.9	−7.8
Glaucine	−6.3	−7.3	−5.0	−7.2	−6.8	−8.1	−6.5	−7.1	−6.5
Laudanosine	−6.4	−7.8	−4.7	−7.2	−6.6	−8.8	−6.9	−8.0	−6.8
Laudanidine	−6.8	−8.3	−4.7	−7.3	−6.6	−6.7	−6.9	−8.2	−7.0
Reticuline	−7.5	−8.8	−5.0	−7.1	−6.7	−8.7	−6.8	−8.3	−7.2
Jacularine	−7.5	−7.9	−5.4	−8.1	−7.2	−7.9	−8.1	−8.3	−7.5
Pronuciferine	−6.3	−8.5	−4.9	−7.2	−6.9	−8.0	−7.0	−7.3	−6.9
Salutaridine	−7.3	−9.0	−4.9	−6.6	−7.0	−7.3	−6.5	−7.1	−6.7
Norsalutaridine	−7.0	−9.3	−4.8	−6.5	−7.0	−7.3	−6.3	−7.4	−6.5
Wilsonirine	−6.9	−8.9	−5.4	−7.7	−6.9	−8.9	−6.6	−7.6	−7.8
Crotonosine	−7.6	−7.8	−5.0	−8.0	−7.2	−7.6	−7.7	−7.8	−7.8
Homolinearisine	−7.7	−8.3	−5.0	−8.1	−7.3	−7.7	−6.9	−7.5	−7.5
Linearisine	−7.3	−7.7	−4.8	−8.1	−7.3	−8.0	−6.7	−8.2	−7.4
Hernovine	−6.7	−7.3	−4.9	−6.8	−6.8	−8.7	−6.3	−6.8	−7.0
N-Methylhernovine	−6.6	−7.6	−4.6	−6.2	−8.3	−8.2	−6.5	−6.1	−6.9
10-O-Methylhernovine	−6.5	−7.4	−5.0	−6.7	−6.6	−8.5	−6.4	−7.0	−7.2
Litseglutine B	−6.6	−7.4	−4.5	−5.8	−8.0	−7.2	−6.4	−6.3	−6.9
Co-crystallized ligand (RMSD, Å)	--	−8.0 (1.458)	--	--	−5.8 (2.385)	−7.9 (1.809)	−11.3 (1.329)	−8.4 (1.383)	−6.4 (2.483)
Pentamidine ^1^	−6.2 ^1^	−7.4	−4.2 ^1^	−7.5 ^1^	--	--	--	--	--

^1^ used as control, RMSD: root mean square deviation.

## Data Availability

The original contributions presented in this study are included in the article/Supplementary Material. Further inquiries can be directed to the corresponding authors.

## Supplementary Materials

The following supporting information can be downloaded at: https://www.mdpi.com/article/10.3390/ph18111715/s1, Table S1: Grid box parameters selected for the target enzymes. Table S2: Human homologous enzymes selected for docking-based selectivity analysis. Table S3: (a): Calculated ΔScore values for alkaloid compounds docked against human homologs (*Trypanosoma* species). (b): Calculated ΔScore values for alkaloid compounds docked against human homologs (*Leishmania* species and *P. falciparum*). Table S4: Main predicted physicochemical, pharmacokinetic, and toxicity-related ADMET parameters for the evaluated alkaloids. Figure S1: Docked structure of the co-crystallized ligand NEE in *T. cruzi* Cyp51 target, describing the amino acid residues of the catalytic site involved in the complex stabilization. Figure S2: Docked structure of the co-crystallized ligand KRM in *T. brucei* ADkinase target, describing the amino acid residues of the catalytic site involved in the complex stabilization. Figure S3: Docked structure of the co-crystallized ligand TPF in *L. infantum* Cyp51 target, describing the amino acid residues of the catalytic site involved in the complex stabilization.

## References

[1] World Health Organization (2024). Global Report on Neglected Tropical Diseases 2024.

[2] El-Shaibany A., Alhakami I., Humaid A., Elasser M. (2023). A review article of pythochemical constitutions of *Croton genus*. Eur. J. Pharm. Med. Res..

[3] Salatino A., Salatino M., Negri G. (2007). Traditional uses, Chemistry and Pharmacology of *Croton species* (*Euphorbiaceae*). J. Braz. Chem. Soc..

[4] McCormack J., Maier K., Wallens P. (2011). Bush Medicine of the Bahamas: A Cross-cultural Perspective from San Salvador Island including Pharmacology and Oral Histories.

[5] Bailey-Shaw Y.A., Salmon C.N., Green C.E., Hibbert S.L., Smith A.M., Williams L.A. (2012). Evaluation of the nutraceutical potential of *Rytidophyllum tomentosum* (L.) Mart.: HPTLC fingerprinting, elemental composition, phenolic content, and in vitro antioxidant activity. Pharm. Crops.

[6] García Díaz J., Tuenter E., Escalona Arranz J.C., Llauradó Maury G., Cos P., Pieters L. (2019). Antimicrobial activity of leaf extracts and isolated constituents of *Croton linearis*. J. Ethnopharmacol..

[7] García-Díaz J., Escalona-Arranz J.C., Ochoa-Pacheco A., Santos S.G.D., González-Fernández R., Rojas-Vargas J.A., Monzote L., Setzer W.N. (2022). Chemical composition and in vitro and in silico antileishmanial evaluation of the essential oil from *Croton linearis* Jacq. stems. Antibiot..

[8] García Díaz J., Tuenter E., Escalona Arranz J.C., Llauradó Maury G., Cos P., Pieters L. (2022). Antiplasmodial activity of alkaloids from *Croton linearis* leaves. Exp. Parasitol..

[9] Al-Snafi A.E. (2024). Antiparasitic activities of medicinal plants: An overview. GSC Biol. Pharm. Sci..

[10] Ogungbe I.V., Setzer W.N. (2016). The Potential of Secondary Metabolites from Plants as Drugs or Leads against Protozoan Neglected Diseases—Part III: In-Silico Molecular Docking Investigations. Molecules.

[11] Mao E.Y., Page S.W., Sleebs B.E., Gancheva M.R., Wilson D.W. (2025). A review of natural products as a source of next-generation drugs against apicomplexan parasites. Npj Antimicrob. Resist..

[12] Haynes L.J., Stuart K.L., Barton D.H.R., Kirby G.W. (1996). Alkaloids from *Croton* species.: Part III. The Constitution of the Proaporphines Crotonosine, “Homolinearisine”, Base A, and the Di-hydroproaporphine Linearisine. Reason and Imagination.

[13] Lepesheva G.I., Hargrove T.Y., Anderson S., Kleshchenko Y., Furtak V., Wawrzak Z., Villalta F., Waterman M.R. (2010). Structural insights into inhibition of sterol 14alpha-demethylase in the human pathogen *Trypanosoma cruzi*. J. Biol. Chem..

[14] Isern J.A., Carlucci R., Labadie G.R., Porta E.O. (2025). Progress and prospects of triazoles in advanced therapies for parasitic diseases. Trop. Med. Infect. Dis..

[15] Beltran-Hortelano I., Alcolea V., Font M., Pérez-Silanes S. (2022). Examination of multiple *Trypanosoma cruzi* targets in a new drug discovery approach for Chagas disease. Bioorganic Med. Chem..

[16] Juárez-Saldivar A., Schroeder M., Salentin S., Haupt V.J., Saavedra E., Vázquez C., Reyes-Espinosa F., Herrera-Mayorga V., Villalobos-Rocha J.C., García-Pérez C.A. (2020). Computational Drug Repositioning for Chagas Disease Using Protein-Ligand Interaction Profiling. Int. J. Mol. Sci..

[17] Panecka-Hofman J., Poehner I., Wade R.C. (2022). Anti-trypanosomatid structure-based drug design—Lessons learned from targeting the folate pathway. Expert. Opin. Drug Discov..

[18] Nardella F., Dobrescu I., Hassan H., Rodrigues F., Thiberge S., Mancio-Silva L., Tafit A., Jallet C., Cadet-Daniel V., Goussin S. (2023). Hemisynthetic alkaloids derived from trilobine are antimalarials with sustained activity in multidrug-resistant *Plasmodium falciparum*. iScience.

[19] Choi J.Y., Calvet C.M., Gunatilleke S.S., Ruiz C., Cameron M.D., McKerrow J.H., Podust L.M., Roush W.R. (2013). Rational Development of 4-Aminopyridyl-Based Inhibitors Targeting *Trypanosoma cruzi* CYP51 as Anti-Chagas Agents. J. Med. Chem..

[20] Kuettel S., Greenwald J., Kostrewa D., Ahmed S., Scapozza L., Perozzo R. (2011). Crystal Structures of T. b. rhodesiense Adenosine Kinase Complexed with Inhibitor and Activator: Implications for Catalysis and Hyperactivation. PLOS Neglected Trop. Dis..

[21] Hofer A. (2023). Targeting the nucleotide metabolism of *Trypanosoma brucei* and other trypanosomatids. FEMS Microbiol. Rev..

[22] Vodnala M., Fijolek A., Rofougaran R., Mosimann M., Mäser P., Hofer A. (2008). Adenosine Kinase Mediates High Affinity Adenosine Salvage in *Trypanosoma brucei*. J. Biol. Chem..

[23] Bessa C.D.P.B., Feu A.E., de Menezes R.P.B., Scotti M.T., Lima J.M.G., Lima M.L., Tempone A.G., de Andrade J.P., Bastida J., Borges W.d.S. (2024). Multitarget anti-parasitic activities of isoquinoline alkaloids isolated from Hippeastrum aulicum (*Amaryllidaceae*). Phytomedicine.

[24] Latham B.D., Geffert R.M., Jackson K.D. (2024). Kinase Inhibitors FDA Approved 2018–2023: Drug Targets, Metabolic Pathways, and Drug-Induced Toxicities. Drug Metab. Dispos..

[25] Tanwar S., Kalra S., Bari V.K. (2024). Insights into the role of sterol metabolism in antifungal drug resistance: A mini-review. Front. Microbiol..

[26] Hargrove T.Y., Wawrzak Z., Liu J., Nes W.D., Waterman M.R., Lepesheva G.I. (2011). Substrate preferences and catalytic parameters determined by structural characteristics of sterol 14α-demethylase (CYP51) from *Leishmania infantum*. J. Biol. Chem..

[27] Panecka-Hofman J., Poehner I. (2023). Structure and dynamics of pteridine reductase 1: The key phenomena relevant to enzyme function and drug design. Eur. Biophys. J..

[28] Singh S., Prajapati V.K. (2022). Exploring actinomycetes natural products to identify potential multi-target inhibitors against *Leishmania donovani*. 3 Biotech..

[29] Pippione A.C., Sainas S., Goyal P., Fritzson I., Cassiano G.C., Giraudo A., Giorgis M., Tavella T.A., Bagnati R., Rolando B. (2019). Hydroxyazole scaffold-based *Plasmodium falciparum* dihydroorotate dehydrogenase inhibitors: Synthesis, biological evaluation and X-ray structural studies. Eur. J. Med. Chem..

[30] Sharma M., Pandey V., Poli G., Tuccinardi T., Lolli M.L., Vyas V.K. (2024). A comprehensive review of synthetic strategies and SAR studies for the discovery of PfDHODH inhibitors as antimalarial agents. Part 1: Triazolopyrimidine, isoxazolopyrimidine and pyrrole-based (DSM) compounds. Bioorganic Chem..

[31] Lipinski C.A., Lombardo F., Dominy B.W., Feeney P.J. (2001). Experimental and computational approaches to estimate solubility and permeability in drug discovery and development settings. Adv. Drug Deliv. Rev..

[32] Daina A., Zoete V. (2016). A BOILED-Egg To Predict Gastrointestinal Absorption and Brain Penetration of Small Molecules. ChemMedChem.

[33] Schinkel A.H. (1999). P-Glycoprotein, a gatekeeper in the blood-brain barrier. Adv. Drug Deliv. Rev..

[34] Bauer M., Tournier N., Langer O. (2019). Imaging P-Glycoprotein Function at the Blood–Brain Barrier as a Determinant of the Variability in Response to Central Nervous System Drugs. Clin. Pharmacol. Ther..

[35] Lee J., Beers J.L., Geffert R.M., Jackson K.D. (2024). A Review of CYP-Mediated Drug Interactions: Mechanisms and In vitro Drug-Drug Interaction Assessment. Biomolecules.

[36] Baell J.B., Holloway G.A. (2010). New substructure filters for removal of pan assay interference compounds (PAINS) from screening libraries and for their exclusion in bioassays. J. Med. Chem..

[37] Daina A., Michielin O., Zoete V. (2017). SwissADME: A free web tool to evaluate pharmacokinetics, drug-likeness and medicinal chemistry friendliness of small molecules. Sci. Rep..

[38] Trott O., Olson A.J. (2010). AutoDock Vina: Improving the speed and accuracy of docking with a new scoring function, efficient optimization and multithreading. J. Comput. Chem..

[39] Evans D.A. (2014). History of the Harvard ChemDraw Project. Angew. Chem. Int. Ed..

[40] Brak K., Kerr I.D., Barrett K.T., Fuchi N., Debnath M., Ang K., Engel J.C., McKerrow J.H., Doyle P.S., Brinen L.S. (2010). Nonpeptidic Tetrafluorophenoxymethyl Ketone Cruzain Inhibitors as Promising New Leads for Chagas Disease Chemotherapy. J. Med. Chem..

[41] Andriani G., Amata E., Beatty J., Clements Z., Coffey B.J., Courtemanche G., Devine W., Erath J., Juda C.E., Wawrzak Z. (2013). Antitrypanosomal lead discovery: Identification of a ligand-efficient inhibitor of *Trypanosoma cruzi* CYP51 and parasite growth. J. Med. Chem..

[42] Senkovich O., Schormann N., Chattopadhyay D. (2009). Structures of dihydrofolate reductase-thymidylate synthase of *Trypanosoma cruzi* in the folate-free state and in complex with two antifolate drugs, trimetrexate and methotrexate. Acta Crystallogr. D Biol. Crystallogr..

[43] Téllez-Valencia A., Olivares-Illana V., Hernández-Santoyo A., Pérez-Montfort R., Costas M., Rodríguez-Romero A., López-Calahorra F., Tuena de Gómez-Puyou M., Gómez-Puyou A. (2004). Inactivation of Triosephosphate Isomerase from *Trypanosoma cruzi* by an Agent that Perturbs its Dimer Interface. J. Mol. Biol..

[44] Kerr I.D., Lee J.H., Farady C.J., Marion R., Rickert M., Sajid M., Pandey K.C., Caffrey C.R., Legac J., Hansell E. (2009). Vinyl Sulfones as Antiparasitic Agents and a Structural Basis for Drug Design. J. Biol. Chem..

[45] Navarro M.V.d.A.S., Gomes Dias S.M., Mello L.V., da Silva Giotto M.T., Gavalda S., Blonski C., Garratt R.C., Rigden D.J. (2007). Structural flexibility in *Trypanosoma brucei* enolase revealed by X-ray crystallography and molecular dynamics. FEBS J..

[46] Baiocco P., Colotti G., Franceschini S., Ilari A. (2009). Molecular Basis of Antimony Treatment in Leishmaniasis. J. Med. Chem..

[47] D’Antonio E.L., Ullman B., Roberts S.C., Dixit U.G., Wilson M.E., Hai Y., Christianson D.W. (2013). Crystal structure of arginase from *Leishmania mexicana* and implications for the inhibition of polyamine biosynthesis in parasitic infections. Arch. Biochem. Biophys..

[48] Souza T.A., Trindade D.M., Tonoli C.C., Santos C.R., Ward R.J., Arni R.K., Oliveira A.H., Murakami M.T. (2011). Molecular adaptability of nucleoside diphosphate kinase b from trypanosomatid parasites: Stability, oligomerization and structural determinants of nucleotide binding. Mol. Biosyst..

[49] Aripirala S., Gonzalez-Pacanowska D., Oldfield E., Kaiser M., Amzel L.M., Gabelli S.B. (2014). Structural and thermodynamic basis of the inhibition of *Leishmania major* farnesyl diphosphate synthase by nitrogen-containing bisphosphonates. Biol. Crystallogr..

[50] Cavazzuti A., Paglietti G., Hunter W.N., Gamarro F., Piras S., Loriga M., Allecca S., Corona P., McLuskey K., Tulloch L. (2008). Discovery of potent pteridine reductase inhibitors to guide antiparasite drug development. Proc. Natl. Acad. Sci. USA.

[51] Prade L., Jones A.F., Boss C., Richard-Bildstein S., Meyer S., Binkert C., Bur D. (2005). X-ray Structure of Plasmepsin II Complexed with a Potent Achiral Inhibitor. J. Biol. Chem..

[52] Sanner M.F. (1999). Python: A programming language for software integration and development. J. Mol. Graph. Model..

[53] Nguewa P.A., Fuertes M.A., Cepeda V., Iborra S., Carrión J., Valladares B., Alonso C., Pérez J.M. (2005). Pentamidine is an antiparasitic and apoptotic drug that selectively modifies ubiquitin. Chem. Biodivers..

[54] Hevener K.E., Zhao W., Ball D.M., Babaoglu K., Qi J., White S.W., Lee R.E. (2009). Validation of Molecular Docking Programs for Virtual Screening against Dihydropteroate Synthase. J. Chem. Inf. Mod..

[55] (2021). BIOVIA Discovery Studio Visualizer.

[56] Moreira B.P., Batista I.C.A., Tavares N.C., Armstrong T., Gava S.G., Torres G.P., Mourão M.M., Falcone F.H. (2022). Docking-Based Virtual Screening Enables Prioritizing Protein Kinase Inhibitors With In vitro Phenotypic Activity Against Schistosoma mansoni. Front. Cell Infect. Microbiol..

