# Supplementary material for: Protozoan Neglected Tropical Diseases (NTDs) Target Inhibition of Alkaloids from Croton linearis Jacq Leaves: A Molecular Docking and ADMET Approach
Source: Pharmaceuticals (Basel). 2025 Nov 12;18(11):1715. doi: 10.3390/ph18111715 (PMC12655485; doi:10.3390/ph18111715)
Supplement: Supplementary file 1 [file pharmaceuticals-18-01715-s001.zip › pharmaceuticals-3861930-supplementary.pdf]

Supplementary Material

Protozoan Neglected Tropical Diseases (NTDs) Target Inhibition of Alkaloids from *Croton linearis* Jacq Leaves: A Molecular Docking and ADMET Approach

Julio A. Rojas-Vargas [1,2,*], Jesús García-Díaz [3], Julio C. Escalona-Arranz [4], Jakub Chlebek [3], Lianet Monzote [5,6], William N. Setzer [6,7,8] and Juan A. Castillo-Garit [9,*]

Table of Contents

**Table S1:** Grid box parameters selected for the target enzymes	**2**
**Table S2:** Human homologous enzymes selected for docking-based selectivity analysis	**3**
**Table S3: (a):** Calculated ΔScore values for alkaloid compounds docked against human homologs (*Trypanosoma* species). **(b):** Calculated ΔScore values for alkaloid compounds docked against human homologs (*Leishmania* species and *P. falciparum*).	**4**
**Table S4:** Main predicted physicochemical, pharmacokinetic, and toxicity-related ADMET parameters for the evaluated alkaloids.	**5**
**Figure S1:** Docked structure of the co-crystallized ligand NEE in *T. cruzi* Cyp51 target, describing the amino acid residues of the catalytic site involved in the complex stabilization.	**6**
**Figure S2:** Docked structure of the co-crystallized ligand KRM in *T. brucei* ADkinase target, describing the amino acid residues of the catalytic site involved in the complex stabilization	**7**
**Figure S3:** Docked structure of the co-crystallized ligand TPF in *L. infantum* Cyp51 target, describing the amino acid residues of the catalytic site involved in the complex stabilization.	**8**

Table S1: Grid box parameters selected for the target enzymes

Protein	Species	Co-crystallized ligand[2]	PDB ID	Resolution (Å)	Center Coordinates		
					x	y	x
Cruzain	*T. cruzi*	KB2	3IUT	1.20	0.879	13.317	-1.389
Cyp 51	*T. cruzi*	NEE	4H6O	2.80	2.8664	25.3701	15.7753
DHFR-TS	*T. cruzi*	2CY	3IRO	2.80	46,09	-1.29	35,63
TIM	*T. cruzi*	BTS	1SUX	2.00	24.094	99.222	63.502
Cyp 51	*T. brucei*	18I	4BJK	2.67	26.642	58.216	26.493
Rhodesain	*T. brucei*	D1R	2P7U	1.65	-8.23	2.30	10.28
Enolase	*T. brucei*	PEP	2PTY	2.00	11.399	29.291	37.716
ADkinase	*T. brucei*	KRM	2XTB	2.80	18.41	-28.57	6.49
ArgI	*L. mexicana*	NP**	4ITY	1.80	15.141	-15.125	-5.4
Cyp 51	*L. infantum*	TPF	3L4D	2.75	31.917	-28.96	-1.658
TryR	*L. infantum*	NP[1]	2JK6	2.95	30.449	47.483	-4.312
NDKb	*L. major*	NP[1]	3NGU	2.29	-15.885	-9.192	-5.732
FPPS	*L. major*	476	4JZX	1.80	56.400	43.880	37.983
PTR1	*L. major*	DVP	3H4V	2.40	-6.132	-12.952	33.845
Plasmepsin2	*P. falciparum*	IH4	2BJU	1.56	-2.625	64.868	9.452
DHODH	*P. falciparum*	DZB	6I55	1.98	16.589	22.366	35.210
Falcipain-3	*P. falciparum*	C1P	3BWK	2.42	6.452	-22.909	50.604

[1]**NP:** no co-crystallized ligand present. [2]Co-crystallized ligands. **KB2:** (3S)-3-(4-((1S)-1,2-dimethyl-1-[(quinolin-6-ylmethyl)amino]propyl)-1H-1,2,3-triazol-1-yl)heptan-2-one. **NEE:** 1-[3-(4-chloro-3,5-dimethylphenoxy)benzyl]-1H-imidazole. **2CY:** 5-[3-(3-fluorophenoxy)propoxy]quinazoline-2,4-diamine. **BTS:** 3-(2-benzothiazolylthio)-1-propanesulfonic acid. **18I:** 3,3'-difluoro-N-[(2S)-3-(1H-indol-3-yl)-1-oxo-1-(pyridin-4-ylamino)propan-2-yl]biphenyl- 4-carboxamide.**D1R:** N-α-[(4-methylpiperazin-1-yl)carbonyl]-N-((1s)-3-phenyl-1-[2-(phenylsulfonyl)ethyl]-propyl)- l-phenylalaninamide. **PEP:** Phosphoenolpyruvate. **KRM:** 4-[5-(4-phenoxyphenyl)-1h-pyrazol-3-yl]morpholine. **TPF:** 2-(2,4-difluorophenyl)-1,3-di(1h-1,2,4-triazol- 1-yl)propan-2-ol. **IH4:** N-(R-carboxy-ethyl)-α-(S)-(2-phenylethyl). **DZB:** N-(2,2-Diphenylethyl)-4-hydroxy-1,2,5-thiadiazole-3-carboxamide. **C1P:** N~2~-(morpholin-4-ylcarbonyl)-N-[(3S)-1-phenyl-5-(phenylsulfonyl)pentan-3-yl]-L-leucinamide. **DVP:** methyl 1-(4-([(2,4-diaminopteridin-6-yl)methyl]amino)benzoyl)piperidine-4-carboxylate. **476:** 3-butyl-1-(2,2-diphosphonoethyl)pyridinium.

Table S2: Human homologous enzymes selected for docking-based selectivity analysis

Parasite protein	Species	PDB ID	Human protein	PDB ID	Resol. (Å)
Cruzain	*T. cruzi*	3IUT			
Rhodesain	*T. brucei*	2P7U	Cathepsin L	8QKB	1.60
Falcipain-3	*P. falciparum*	3BWK			
Cyp 51	*T. cruzi*	4H6O			
Cyp 51	*T. brucei*	4BJK	Cyp51A1	3LD6	2.80
Cyp 51	*L. infantum*	3L4D			
DHFR-TS	*T. cruzi*	3IRO	DHFR	1DRF	2.00
PTR1*	*L. major*	3H4V			
DHFR-TS	*T. cruzi*	3IRO	TS	1HVY	1.90
Enolase	*T. brucei*	2PTY	Enolase	2PTY	2.00
ADkinase	*T. brucei*	2XTB	ADkinase	2I6B	2.30
TryR	*L. infantum*	2JK6	Glutathione reductase	1XAN	2.00
ArgI	*L. mexicana*	4ITY	ArgI	3MFV	1.90
NDKb	*L. major*	3NGU	NDKb	2HVD	2.15
FPPS	*L. major*	4JZX	FPPS	5DGN	2.08
Plasmepsin2	*P. falciparum*	2BJU	Cathepsin D	4OBZ	2.90
DHODH	*P. falciparum*	6I55	DHODH	4RR4	2.38
TIM	*T. cruzi*	1SUX	TIM	4POC	1.60

*There is no human counterpart; the closest is human DHFR. The absence of PTR1 in humans represents an advantage in terms of pharmacological specificity.

Table S3 (a): Calculated ΔScore values for alkaloid compounds docked against human homologs (*Trypanosoma* species). **(b):** Calculated ΔScore values for alkaloid compounds docked against human homologs (*Leishmania* species and *P. falciparum*).

(a)

**Alkaloids**	**Human protein**								
	Trypanosoma cruzi					***Trypanosoma brucei***			
	Cathepsin L	TIM	Cyp51A1	TS	DHFS	Cyp51A1	Cathepsin L	ADkinase	Enolase
Corydine	-0.4	0.3	-1.3	-0.1	-0.1	-0.5	-0.8	0.4	0.3
Cularine	-0.1	0.9	-1.3	0.2	-0.2	-1	-0.1	0.2	0.1
Glaucine	-0.4	0.9	-1.6	0.5	-0.5	-0.7	0.4	-0.3	0.2
Laudanosine	0.8	0.2	-1.8	-0.4	-0.4	-0.4	0.8	0.3	0.9
Laudanidine	1.2	-0.2	-0.6	0.2	-0.5	-0.3	1.0	0	0.9
Reticuline	1.3	0.7	-0.9	-0.3	-0.5	-1.0	1.0	0.3	0.5
Jacularine	-0.3	0.9	-1.4	0	-1.0	-0.7	0.4	-1.1	-0.2
Pronuciferine	-0.3	1.3	-1.8	-0.2	-1.1	-0.2	-0.2	-0.6	0.4
Salutaridine	-0.7	1	-1.2	0.6	-0.9	-0.1	-0.2	0.5	0.7
Norsalutaridine	-0.6	0.4	-0.4	0.5	0.1	-0.1	-0.2	0.4	0.7
Wilsonirine	0.3	1.1	-1.2	0.1	0.2	-0.5	0.8	-0.6	0.7
Crotonosine	0.3	1.1	-1.1	0.1	-1.0	-0.7	0.4	-0.1	-0.3
Homolinearisine	0.4	0.6	-0.7	-0.1	-0.7	-0.6	0.3	0.3	0.1
Linearisine	0.1	1.1	-0.7	0	-1.2	-0.8	0.2	-0.4	0
Hernovine	-1	0.8	-0.8	-0.3	-0.3	-0.3	-0.8	0.1	0.2
N-Methylhernovine	0.1	0.4	-1.9	0.4	-1.0	-1.0	0.4	0.3	1.1
10-O-Methylhernovine	-0.6	0.8	-1.3	-0.1	-0.1	-0.5	-0.6	-0.1	0.4
Litseglutine B	-0.1	2.0	-1.9	0.3	-1.4	-0.9	0.1	0.2	1.2

(b)

**Alkaloids**	**Human protein**							
	L. major		***L.mexicana***	***L. infantum***		***P. falcisparum***		
	FPPS	NDKb	ArgI	Glutathione	Cyp51A1	DHODH	CapthesinD	CapthesinL
Corydine	0.5	-1.5	0.3	-1.7	-1.9	0.8	-0.3	0.2
Cularine	0.5	-0.7	0.3	-1.4	-1	0.5	-0.9	1.4
Glaucine	0.4	-0.2	0.2	-1.3	-1.5	0	-0.1	0.3
Laudanosine	-0.1	-0.3	-0.3	-1.8	-0.9	0	-0.3	1
Laudanidine	-0.4	-0.4	0.5	-2.2	-0.4	-0.2	-0.1	1.1
Reticuline	-0.4	-0.5	0.8	-2.1	-0.4	1	-0.4	1.1
Jacularine	0.4	0.1	0.4	-0.9	-1.5	-0.3	0.7	0.8
Pronuciferine	0	-0.4	-0.2	-1.6	-0.8	-0.2	0.3	0.5
Salutaridine	0	0	0.1	-2	-0.1	-0.4	-1	-0.2
Norsalutaridine	0	-0.2	0.3	-1.6	0.1	0	-1.1	-0.4
Wilsonirine	0.3	-0.4	-0.3	-1	0	0.3	-0.5	1.6
Crotonosine	0.7	0	0.2	-1.3	-1.4	0.3	1	1.3
Homolinearisine	0.6	0	1	-1.6	-0.6	-0.1	-0.3	1
Linearisine	0.7	0.2	0.9	-2.1	-1.3	1.4	-0.6	0.6
Hernovine	0.3	-0.6	0.1	-1.1	-1.4	0.5	-0.5	0.2
N-Methylhernovine	2.1	-0.2	-0.6	-1.7	-1.6	-1.1	0	1.1
10-O-Methylhernovine	-0.1	-0.7	0.6	-1.1	-1.5	0.5	-0.7	0.5
Litseglutine B	1.6	-0.7	0.5	-2	-1.7	-0.3	-0.1	0.9

Table S4: Main predicted physicochemical, pharmacokinetic, and toxicity-related ADMET parameters for the evaluated alkaloids.

Alkaloids	MW	iLOGP	TPSA	Pgp substrate	CYP1A2 inhibitor	CYP2C9 inhibitor	CYP2C19 inhibitor	CYP2D6 inhibitor	CYP3A4 inhibitor
Corydine	341.4	3.37	51.16	Yes	Yes	No	No	Yes	Yes
Cularine	341.4	3.58	40.16	Yes	Yes	No	Yes	Yes	Yes
Glaucine	355.43	3.72	40.16	Yes	No	No	No	Yes	Yes
Laudanosine	357.44	3.65	40.16	No	No	No	No	Yes	Yes
Laudanidine	343.42	3.48	51.16	No	No	No	No	Yes	Yes
Reticuline	329.39	3.13	62.16	Yes	No	No	No	Yes	No
Jacularine	285.34	2.45	58.56	Yes	No	No	No	Yes	No
Pronuciferine	311.37	2.94	38.77	No	Yes	Yes	No	Yes	Yes
Salutaridine	327.37	2.74	59	Yes	No	No	No	No	No
Norsalutaridine	313.35	2.5	67.79	Yes	No	No	No	No	No
Wilsonirine	327.37	3.18	59.95	Yes	Yes	No	No	Yes	Yes
Crotonosine	283.32	2.26	58.56	Yes	Yes	No	No	Yes	No
Homolinearisine	297.35	2.53	49.77	Yes	Yes	Yes	No	Yes	No
Linearisine	299.36	2.56	49.77	Yes	No	No	No	Yes	No
Hernovine	313.35	2.78	70.95	Yes	Yes	No	No	Yes	Yes
N-Methylhernovine	327.37	3.17	62.16	Yes	Yes	No	No	Yes	Yes
10-O-methylhernovine	327.37	3.07	59.95	Yes	Yes	No	No	Yes	Yes
Litseglutine B	341.4	3.38	51.16	Yes	Yes	No	No	Yes	Yes

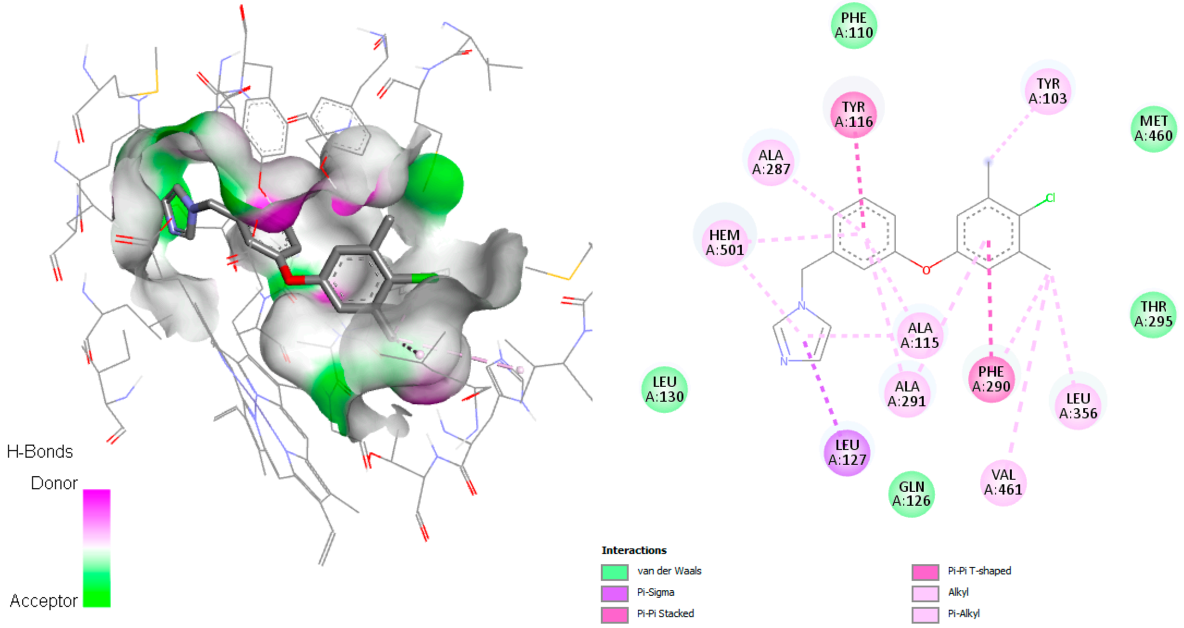

Figure S1: Docked structure of the co-crystallized ligand NEE in *T. cruzi* Cyp51 target, describing the amino acid residues of the catalytic site involved in the complex stabilization.

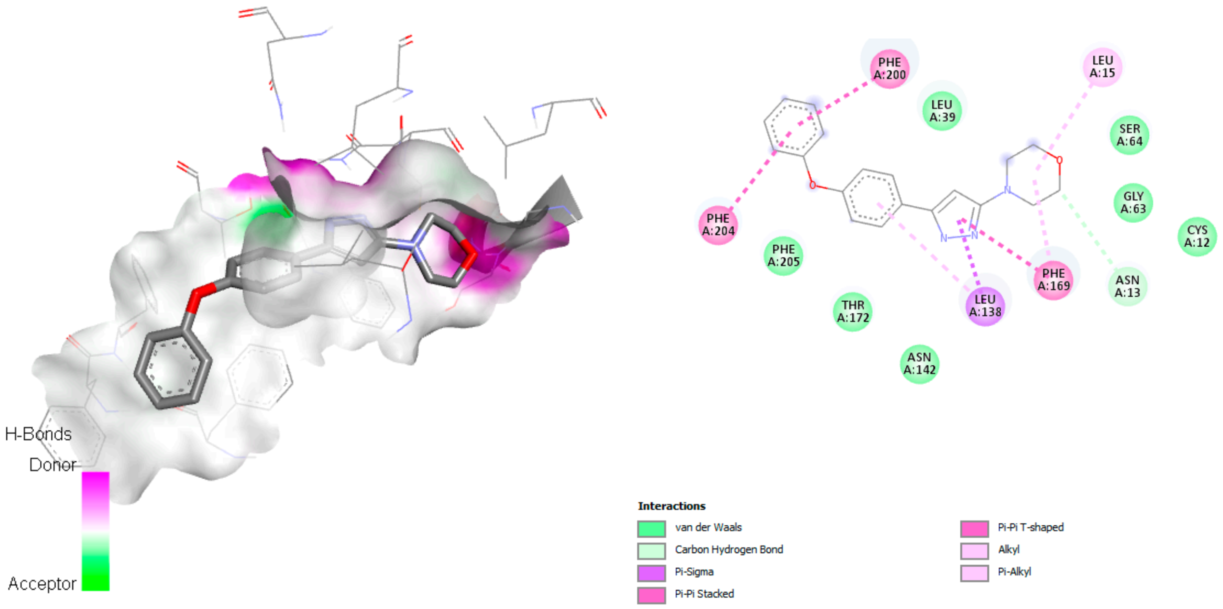

Figure S2: Docked structure of the co-crystallized ligand KRM in *T. brucei* ADkinase target, describing the amino acid residues of the catalytic site involved in the complex stabilization.

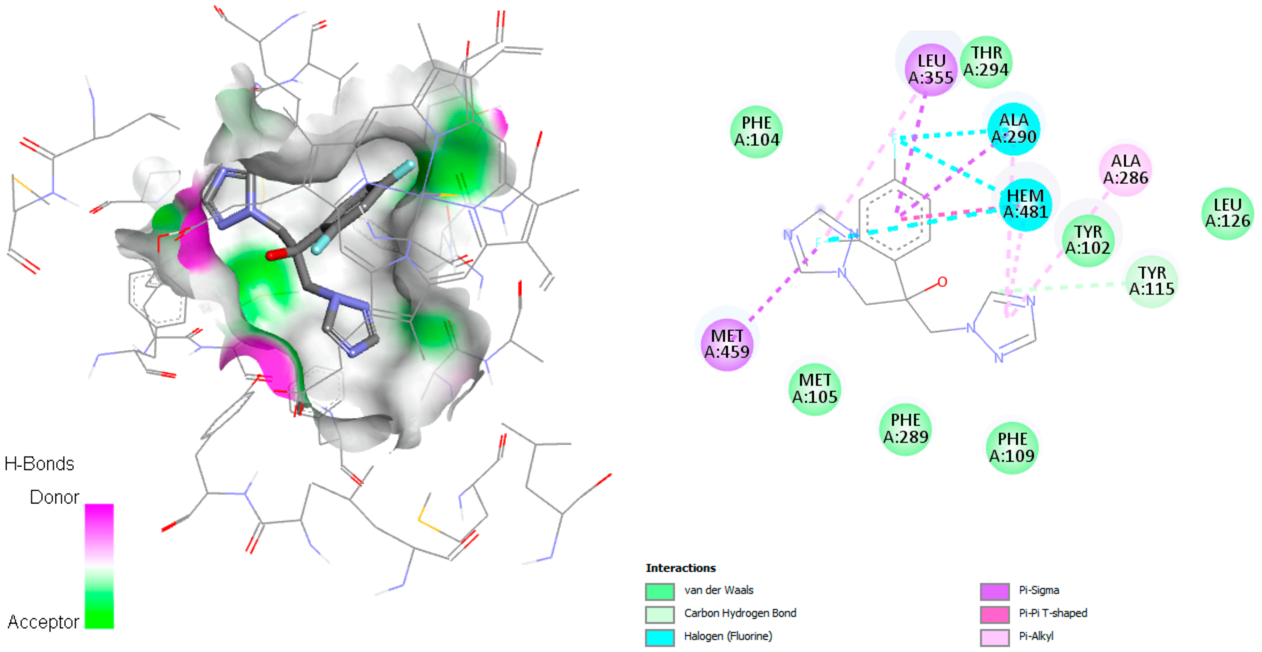

Figure S3: Docked structure of the co-crystallized ligand TPF in *L. infantum* Cyp51 target, describing the amino acid residues of the catalytic site involved in the complex stabilization.
